# CDK2 inhibitors as candidate therapeutics for cisplatin- and noise-induced hearing loss

**DOI:** 10.1084/jem.20172246

**Published:** 2018-04-02

**Authors:** Tal Teitz, Jie Fang, Asli N. Goktug, Justine D. Bonga, Shiyong Diao, Robert A. Hazlitt, Luigi Iconaru, Marie Morfouace, Duane Currier, Yinmei Zhou, Robyn A. Umans, Michael R. Taylor, Cheng Cheng, Jaeki Min, Burgess Freeman, Junmin Peng, Martine F. Roussel, Richard Kriwacki, R. Kiplin Guy, Taosheng Chen, Jian Zuo

**Affiliations:** 1 Department of Developmental Neurobiology, St. Jude Children’s Research Hospital, Memphis, TN; 2 Department of Chemical Biology and Therapeutics, St. Jude Children’s Research Hospital, Memphis, TN; 3 Department of Structural Biology, St. Jude Children’s Research Hospital, Memphis, TN; 4 Department of Tumor Cell Biology, St. Jude Children’s Research Hospital, Memphis, TN; 5 Department of Biostatistics, St. Jude Children’s Research Hospital, Memphis, TN; 6 Preclinical PK Shared Resource, St. Jude Children’s Research Hospital, Memphis, TN

## Abstract

Hearing loss caused by aging, noise, cisplatin toxicity, or other insults affects 360 million people worldwide, but there are no Food and Drug Administration–approved drugs to prevent or treat it. We screened 4,385 small molecules in a cochlear cell line and identified 10 compounds that protected against cisplatin toxicity in mouse cochlear explants. Among them, kenpaullone, an inhibitor of multiple kinases, including cyclin-dependent kinase 2 (CDK2), protected zebrafish lateral-line neuromasts from cisplatin toxicity and, when delivered locally, protected adult mice and rats against cisplatin- and noise-induced hearing loss. CDK2-deficient mice displayed enhanced resistance to cisplatin toxicity in cochlear explants and to cisplatin- and noise-induced hearing loss in vivo. Mechanistically, we showed that kenpaullone directly inhibits CDK2 kinase activity and reduces cisplatin-induced mitochondrial production of reactive oxygen species, thereby enhancing cell survival. Our experiments have revealed the proapoptotic function of CDK2 in postmitotic cochlear cells and have identified promising therapeutics for preventing hearing loss.

## Abstract (precis)

Using a high-throughput small molecule screen, Teitz et al. identify kenpaullone, a cyclin-dependent kinase 2 inhibitor, which when delivered locally confers protection against cisplatin- and noise-induced hearing loss in zebrafish, mice, and rats and reduces mitochondrial ROS production and cochlear cell death.

## Introduction

More than 360 million people worldwide suffer from hearing loss caused by noise, chemotherapy, antibiotics, viral infections, genetic predisposition, or aging (World Health Organization, 2017). Cisplatin is a widely used chemotherapeutic agent, but one of its major side effects is irreversible sensorineural hearing loss, which occurs in 50–70% of patients with cancer treated with cisplatin (Fouladi et al., 2008; Knight et al., 2017). Recently, genomic loci have been identified that predispose pediatric patients with brain tumors to hearing loss when treated with cisplatin (Ross et al., 2009; Xu et al., 2015). These genomic loci can help identify the specific patients to whom the protective drugs should be given, thus individualizing the treatment. Noise can induce stress in cochlear cells and cause damage to the connecting nerves, resulting in transient or permanent hearing loss, and is a major hazard in civilian and military settings, and age-related hearing loss affects more than half of people older than 75 yr (Liberman, 2015).

There are no Food and Drug Administration (FDA)–approved drugs that protect against noise-, cisplatin-, or antibiotic-induced or age-related hearing loss (Oishi and Schacht, 2011; El Kechai et al., 2015; Müller and Barr-Gillespie, 2015). Despite extensive research, most candidate compounds currently in preclinical or clinical trials are related to antioxidant, vitamin, or glutathione metabolism, and their effectiveness remains unclear (Rybak and Ramkumar, 2007; Forge and Van De Water, 2008; Tieu and Campbell, 2013; Hazlitt et al., 2018). In clinical use, otoprotectants should reduce hearing loss by at least 20 dB at a given frequency or at least 10 dB at any two adjacent frequencies (Campbell et al., 2016). In zebrafish lateral lines, the neuromasts consist of hair cells (HCs) that are also subject to cisplatin and antibiotic toxicity, a feature that has been exploited successfully for in vivo screening of protective compounds (Coffin et al., 2010); however, the effectiveness of the compounds identified in this model has yet to be validated in mammals.

We developed an approach that exploits the mechanistic similarities of noise, antibiotics, aging, and cisplatin in inducing mammalian cochlear cell death. Using an immortalized cell line derived from neonatal mouse cochleae, we performed an unbiased, high-throughput screen (HTS) and identified small molecules that protected against cisplatin ototoxicity. We evaluated our top-hit compounds, including kenpaullone, an inhibitor of cyclin-dependent kinase 2 (CDK2) and other kinases, ex vivo in mouse cochlear explants and in vivo in zebrafish, adult mice, and rats, for protective effects against cisplatin- and noise-induced damage. We further confirmed the mechanisms of action of kenpaullone by analyzing CDK2-deficient mice. Our experiments have revealed the proapoptotic role of CDK2 in postmitotic cochlear cells and have identified a promising preventive treatment for cisplatin- and noise-induced hearing loss.

## Results

### CDK2 inhibitors were among the top hits in the small molecule screen

**Figure 1.**
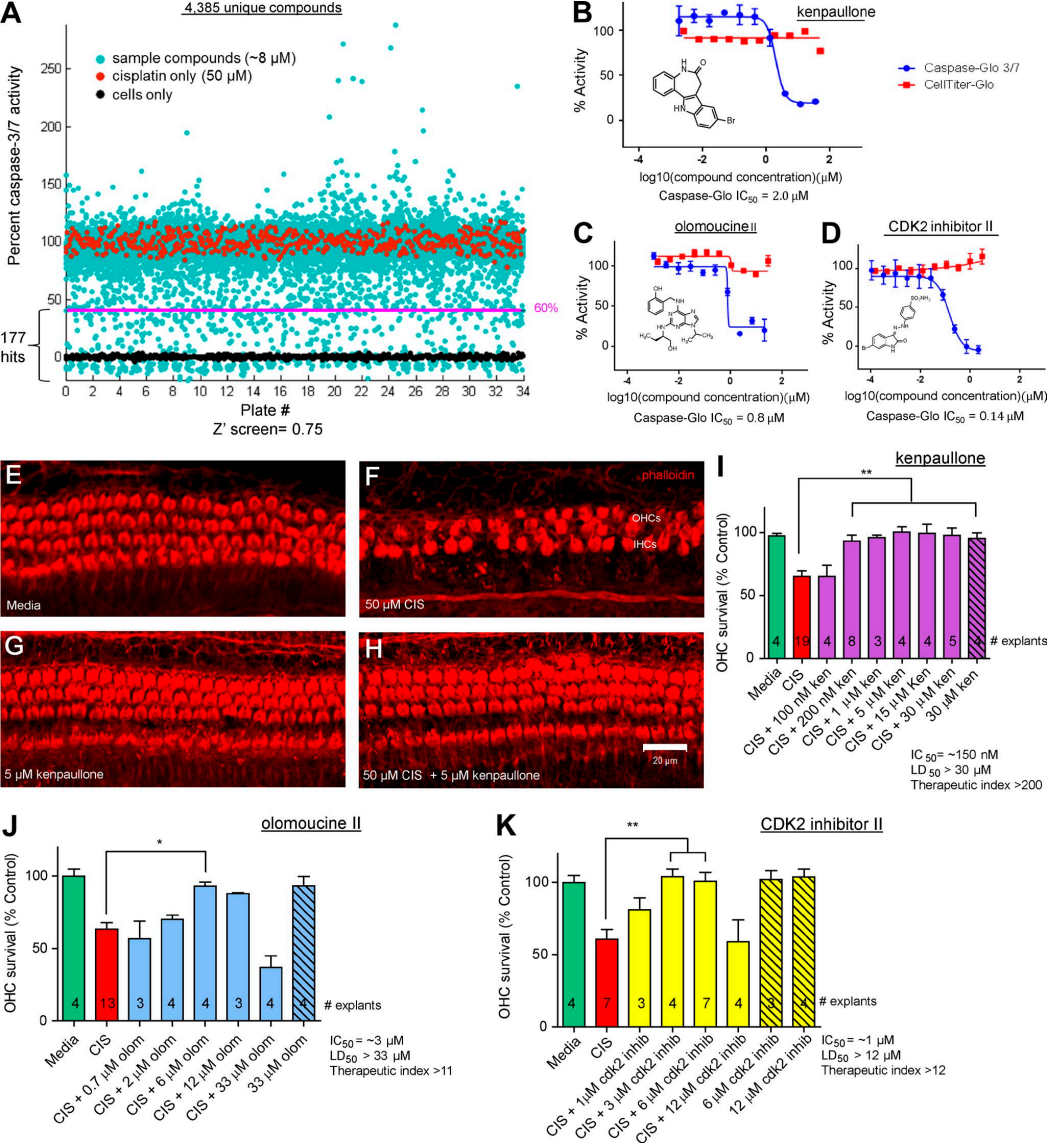
**Screening and identification of kenpaullone and CDK2 inhibitors that protect against cisplatin toxicity in HEI-OC1 cells. (A)** Screening of a bioactive compound library of 4,385 unique compounds, including 845 FDA-approved drugs, in HEI-OC1 cells. Cells treated with 50 µM cisplatin (red dots) were assigned 100% caspase-3/7 activity. Cells not treated with cisplatin, i.e., those grown in medium only (black dots) were assigned 0% caspase-3/7 activity. Each compound was added to a final concentration of 8 µM in the presence of 50 µM cisplatin (cyan dots). The cell-based screen mean Z′ was 0.75, the signal window was 12, and the signal fold change was 4.9. In all, 177 compounds (those on the pink line or below it) were found to decrease cisplatin-induced caspase-3/7 activity by 60% or more and were further analyzed for dose response and toxicity. **(B–D)** Dose–response curves for kenpaullone (B), olomoucine II (C), and CDK2 inhibitor II (D), determined using the Caspase-Glo 3/7 assay (compound + 50 µM cisplatin, blue curves) and the CellTiter-Glo assay for cell viability (compound only, red curves) after incubation for 22 h in culture in HEI-OC1 cells. The caspase-3/7 IC_50_ activity and structure are indicated for each compound. Error bars: SEM. **(E–K)** Kenpaullone (ken; E–I), olomoucine II (olom; J), and CDK2 inhibitor II (K) protected against cisplatin-induced HC loss in mouse cochlear explants. Confocal images of whole-mount middle turn cochlear explants that were treated with medium alone (E), 50 µM cisplatin (F), 5 µM kenpaullone (G), or 50 µM cisplatin and 5 µM kenpaullone (H) for 24 h are shown. Phalloidin labels the HCs. (I–K) OHC survival (percentage; mean ± SEM) after treatment with various concentrations of compounds and/or cisplatin (CIS). The number of cochlear explants analyzed is indicated in each bar. *, P < 0.05 and **, P < 0.01 by one-way ANOVA followed by a Bonferroni comparison.

We used an immortalized cell line (HEI-OC1) derived from mouse cochleae (postnatal day 7 [P7]; Kalinec et al., 2003) to conduct an unbiased screen for compounds protective against cisplatin ototoxicity (Teitz et al., 2016). We screened a bioactive library of 4,385 unique compounds, including 845 FDA-approved drugs (Morfouace et al., 2014) at a concentration of 8 µM, cotreating the cells with 50 µM cisplatin (Fig. 1 A; see dose responses in Fig. S1, B and C). Caspase-3/7 activity was chosen as the endpoint for measuring cell death in an assay that quantifies a luminescent product derived by the specific cleavage of a caspase-3/7 substrate (Caspase-Glo 3/7 reagent; Fig. S1 A); caspase-3/7 activity was defined as 100% in the cells treated with cisplatin alone and as 0% in cells not treated with cisplatin (Fig. 1 A).

We identified 177 hits that reduced cisplatin-induced caspase-3/7 activity by 60% or more. These compounds were further validated and characterized by dose-responsive analysis for potency and toxicity in HEI-OC1 cells; toxicity was assessed by a cell viability assay (CellTiter-Glo) with the test compounds alone (Fig. 1, B–D; and Fig. S1, E–K). The top 10 hits in this screen (with IC_50_ values in the HEI-OC1 cells of 0.04–6.2 µM) and their protective properties, as investigated in this study, are summarized in Table S1. The FDA has approved three of the compounds (leflunomide, cyanocobalamin, and olsalazine sodium) for treating other medical conditions. Strikingly, 3 of the top 10 protective compounds (kenpaullone, olomoucine II, and CDK2 inhibitor II) target CDK2, a canonical cell cycle–promoting protein (Fig. 1, B–D).

### Top hits protected against cisplatin-induced HC death in mouse cochlear explants

Cochlear explants are a widely accepted surrogate for in vivo cochlear models (Masuda et al., 2012). The top 10 hits in our screen were evaluated on P3–P4 mouse cochlear explants (from a 129SvEv/C57BL/6 mixed background; see Fig. S1 [I and J] for the cisplatin dose responses), and all exhibited protection at 20 µM or less (Table S1). Dose–response experiments were conducted for the three CDK2 inhibitor compounds, kenpaullone (Fig. 1, E–I), olomoucine II (Fig. 1 J), and CDK2 inhibitor II (Fig. 1 K), and the IC_50_ values for these compounds against cisplatin were 0.15, 3, and 1 µM, respectively. Of these three CDK2 inhibitors, kenpaullone exhibited the best profile, based on its protective activity in explants and therapeutic index (the LD_50_/IC_50_ ratio), which exceeded 200.

To benchmark kenpaullone to four other compounds currently in clinical trials, we determined the IC_50_ of kenpaullone and these four compounds for protection against cisplatin ototoxicity in cochlear explants under identical conditions. Interestingly, kenpaullone had an IC_50_ of 0.2 µM, whereas d-methionine, ebselen, sodium thiosulfate, and dexamethasone had IC_50_ values of 98, 11, 2, and >0.25 µM, respectively (Fig. S2, A–F), consistent with a previous study on d-methionine and ebselen (Kopke et al., 1997). Kenpaullone outperformed the four benchmark compounds in terms of potency and therapeutic index.

To ascertain whether these top compounds interfered with the tumor-killing ability of cisplatin, we tested them in five tumor cell lines: three tumorsphere lines derived from mouse medulloblastoma (MB; Morfouace et al., 2014) and two human neuroblastoma (NB) cell lines (Barbieri et al., 2006; Altun et al., 2010). When cotreated with cisplatin in vitro, most of the compounds had minimal to no adverse effect on the tumor-killing effects of cisplatin; kenpaullone had some effect in two lines, making it more suitable for local than systemic delivery in mammalian tumor models (Fig. S2 and Table S1). Given these promising properties of kenpaullone, we further tested its protective effects in zebrafish, mice, and rats in vivo.

### Kenpaullone protects against cisplatin-induced HC loss in zebrafish in vivo

**Figure 2.**
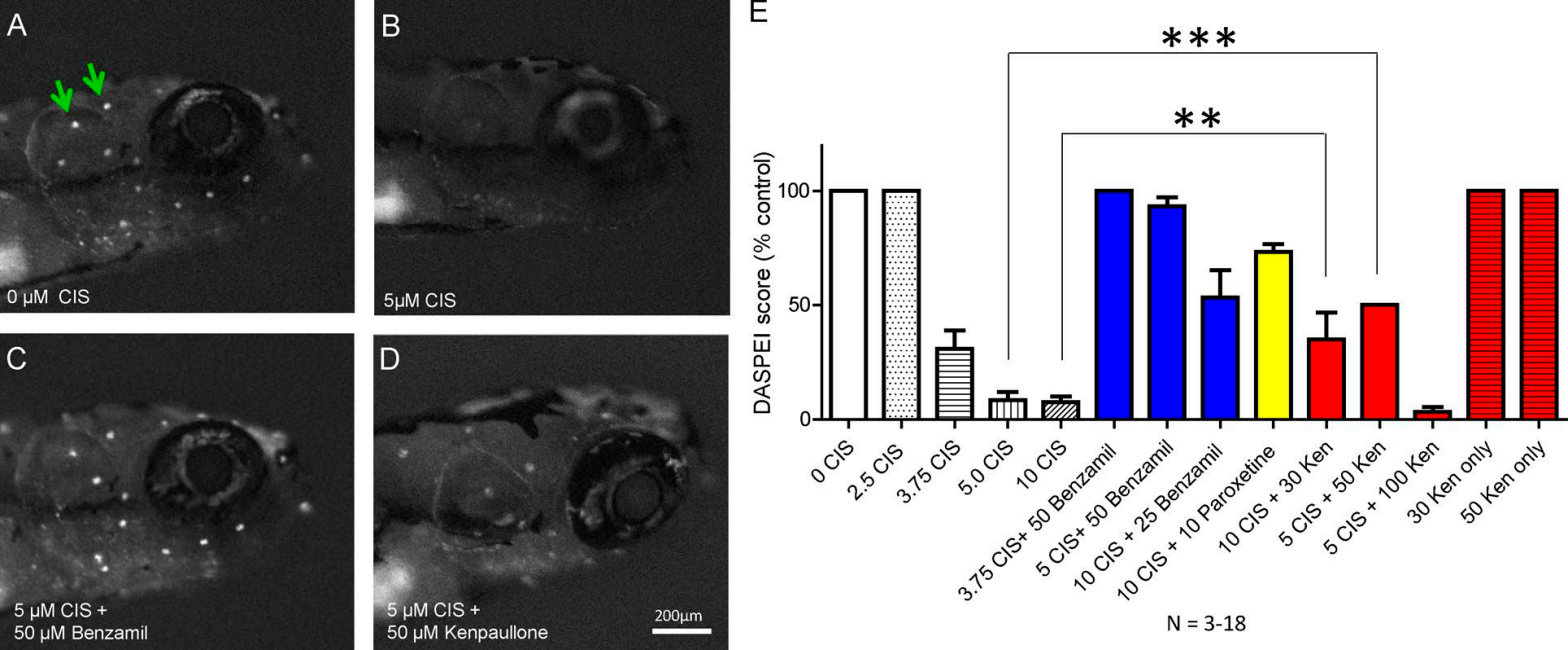
**Kenpaullone protects against cisplatin-induced HC loss in zebrafish lateral lines in vivo**. **(A–D)** Neuromasts (white dots; green arrows) in the zebrafish head were visualized under identical microscopic conditions by staining with 0.005% DASPEI vital dye after treatment for 20 h with medium alone (A), 5 µM cisplatin (B), 5 µM cisplatin and 50 µM benzamil (C), or 5 µM cisplatin and 50 µM kenpaullone. **(E)** The DASPEI intensity score (mean ± SEM) with various concentrations (micromolar) of kenpaullone (Ken), benzamil, paroxetine, and cisplatin (CIS). Benzamil and paroxetine protect against cisplatin-induced HC loss in zebrafish (Vlasits et al., 2012). The number of zebrafish tested for each condition ranged from three to 13. ** P, < 0.01 and *** P, < 0.001 by one-way ANOVA followed by a Bonferroni comparison.

The lateral-line neuromasts of zebrafish are a valuable model for testing compounds protective against cisplatin toxicity in vivo, as their HCs are considered homologous to those in the mammalian inner ear and are readily accessible to drugs in vivo. As in a previous study (Vlasits et al., 2012), we examined 10 neuromasts in zebrafish larvae of the *AB WT strain at 5 d after fertilization for evidence of protection by kenpaullone or one of the other top nine compounds in our screen. The dose response with cisplatin was established (Fig. 2), and the vitality of the neuromast cells was determined by staining with the mitochondrial dye DASPEI (Vlasits et al., 2012). Treatment with 5 µM cisplatin in 0.2% DMSO for 20 h reduced the DASPEI intensity score in zebrafish by 91% (Fig. 2, A–E). Paroxetine and benzamil were used at concentrations (10 and 50 µM, respectively) previously shown to confer protection against cisplatin in zebrafish (Vlasits et al., 2012). Kenpaullone administered at a concentration of 30–50 µM gave 30–50% protection, whereas none of the other nine compounds protected zebrafish neuromasts against cisplatin toxicity under the conditions tested (Table S1). These results show that kenpaullone protects against cisplatin ototoxicity in vivo in nonmammalian vertebrates such as zebrafish.

### Kenpaullone ameliorates cisplatin-induced hearing loss in adult mice and rats in vivo

Because kenpaullone exhibited the best therapeutic index in cochlear explants and is protective in zebrafish neuromasts in vivo, we next tested this compound in adult mice. We used trans-tympanic injection for compound delivery because this approach is well established for treating middle and inner ear diseases in animal models and in clinical trials and can avoid the systemic toxicity of many compounds (El Kechai et al., 2015; Freyer et al., 2017). The maximal nontoxic concentration of kenpaullone injected trans-tympanically was determined in vivo by testing the cochlear toxicity of kenpaullone alone in a series of concentrations (Fig. S3, A–H′). Mice injected with kenpaullone alone at 250 µM showed no toxicity 3 mo after injection (Fig. S3, I–K).

To test whether kenpaullone protected against cisplatin-induced HC and hearing loss in vivo, we also established the cisplatin dose responses for hearing loss in WT FVB adult mice by performing i.p. injections with an optimal cisplatin dose of 30 mg/kg that resulted in threshold elevations of 20–40 dB (Fig. S3, L and M).

**Figure 3.**
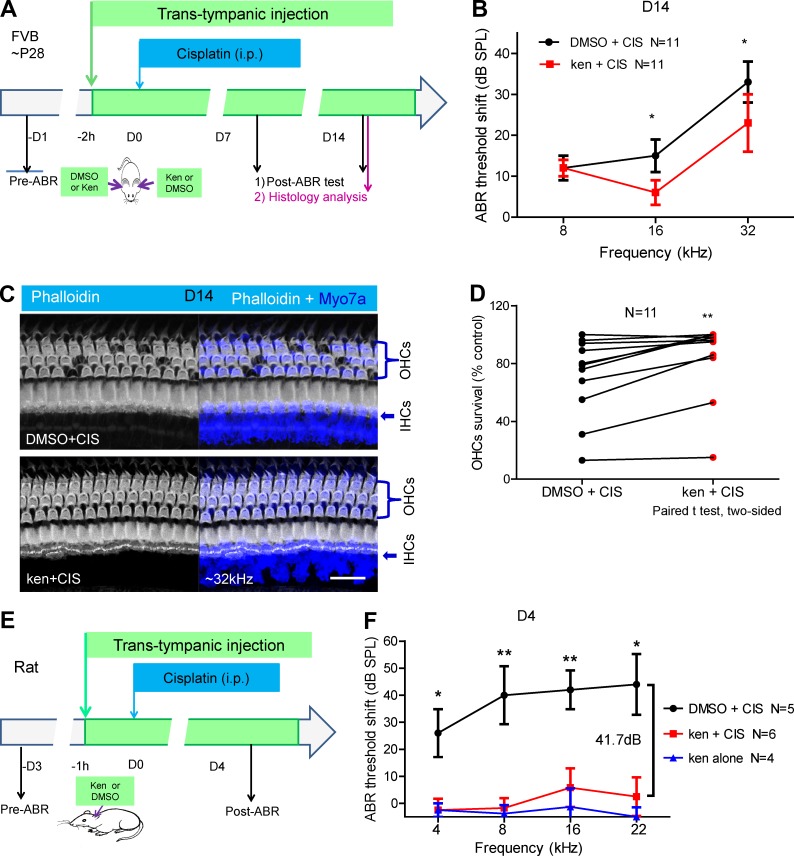
**Locally delivered kenpaullone (ken) protects against cisplatin (CIS)-induced HC loss and hearing loss in adult mice and rats in vivo. (A)** Experimental design. One ear of each FVB WT mouse at P28 was trans-tympanically injected with compound (250 µM ken in 0.5% DMSO in a volume of 5 µl), and the other ear was injected with 0.5% DMSO only, in a random order. 2 h later, the mice were injected i.p. with CIS at 30 mg/kg body weight, which was expected to damage the OHCs in both ears equally. ABR thresholds were recorded for each ear before or at D7 or D14 after ken or DMSO treatment with CIS administration, and cochlear histology was examined at D14. All analysis was performed in a double-blinded manner. **(B)** ABR threshold shifts in ken-treated ears (red) versus DMSO-treated control ears (black) of 11 mice at D14. Results are presented as the mean ± SEM. P = 0.0221 at 16 kHz, and P = 0.0215 at 32 kHz. **(C)** Representative confocal images of cochleae at the 32-kHz region double stained with phalloidin with an antibody to the HC marker myosin 7a (Myo7a) at D14. Bar, 20 µM. **(D)** Comparison of the percentage of OHC survival in the 32-kHz region in ken- and DMSO-treated ears after CIS administration in all 11 mice in four cohorts. Each line links the two cochleae of one mouse. P = 0.0028 by the paired two-tailed Student’s *t* test. **(E and F)** The left ear of each Wistar rat (body weight 306–354 g) was trans-tympanically injected with compound (310 µM ken in 0.5% DMSO; red curve) or carrier (0.5% DMSO; black curve) in a volume of 30 µl. 1 h later, the rat was injected i.p. with CIS at 13 mg/kg body weight. ABR thresholds were recorded 1 d before and 4 d after CIS administration. Error bars: SEM. N, number of rats tested in each group (two cohorts). As controls, four rats were injected trans-tympanically with 310 µM ken only, without CIS treatment (blue curve). All analysis was performed in a double-blinded manner. Note that significant differences in protection (28.5, 41.7, 36.2, and 41.5 dB at 4, 8, 16, and 22 kHz, respectively) were detected between ken/CIS and DMSO/CIS rats, whereas no significant differences were detected between ken/CIS and ken alone rats at these four frequencies. *, P < 0.05 and **, P < 0.01 by the unpaired two-tailed Student’s *t* test.

We then injected adult (P28) FVB mice trans-tympanically with 250 µM kenpaullone in one ear and carrier solution (0.5% DMSO) in the opposite ear, and 2 h later, we administered 30 mg/kg cisplatin i.p. so that it would affect both ears (Fig. 3 A). The thresholds of auditory brainstem responses (ABRs) were measured for each ear at 7 and 14 d (D7 and D14, respectively) after drug exposure (DMSO+cisplatin or kenpaullone+cisplatin). At D7, both ears exhibited similar ABR threshold shifts (Fig. S3 N). However, at D14, DMSO-injected ears showed the expected ABR threshold elevation at all three frequencies (8, 16, and 32 kHz), whereas kenpaullone-injected ears exhibited a significantly reduced ABR threshold elevation, with a mean reduction of ∼10 dB at frequencies of 16 or 32 kHz (Fig. 3 B). Note that the elevations of the DMSO-injected ears are consistent with those in a previous study for FVB mice (Ciarimboli et al., 2010).

Morphological analysis of the cochleae showed that, at D14 after drug exposure, there was a loss of outer HCs (OHCs) at basal turns in DMSO-injected ears but a reduced loss of OHCs in kenpaullone-injected ears (P = 0.0028; Fig. 3, C and D). The OHC loss in DMSO-injected ears was limited mostly to basal turns. Therefore, both ABR hearing tests and OHC morphological measurements demonstrated that kenpaullone, delivered trans-tympanically, ameliorates cisplatin-induced hearing loss under the stated conditions in adult mice.

To overcome the limitations of mouse models of cisplatin ototoxicity and to test the protective effect of kenpaullone against cisplatin ototoxicity in a second mammalian species, we chose the Wistar rat, a well-established outbred rodent strain that exhibits cisplatin-induced hearing loss that is more severe than that seen in inbred mouse strains (Campbell et al., 1996). When 310 µM kenpaullone alone was delivered trans-tympanically, it induced no hearing loss. i.p. treatment with 13 or 16 mg/kg cisplatin and local injection of carrier (0.5% DMSO) alone resulted in hearing loss of 24–44 dB at D4 after cisplatin treatment, which was much worse than that detected in mice. Locally delivered 310 µM kenpaullone provided complete protection against cisplatin ototoxicity at three or four frequencies of 4, 8, 16, and 22 kHz (Fig. 3, E and F; and Fig. S3, O and P), which was not significantly different from the results in rats treated with kenpaullone alone without cisplatin. The differences in protection between DMSO- and kenpaullone-treated rats were robust, reaching up to 41.7 dB at multiple frequencies. These results provide strong evidence that kenpaullone is a potent otoprotectant against cisplatin.

### Kenpaullone ameliorates noise-induced hearing loss in adult mice in vivo

**Figure 4.**
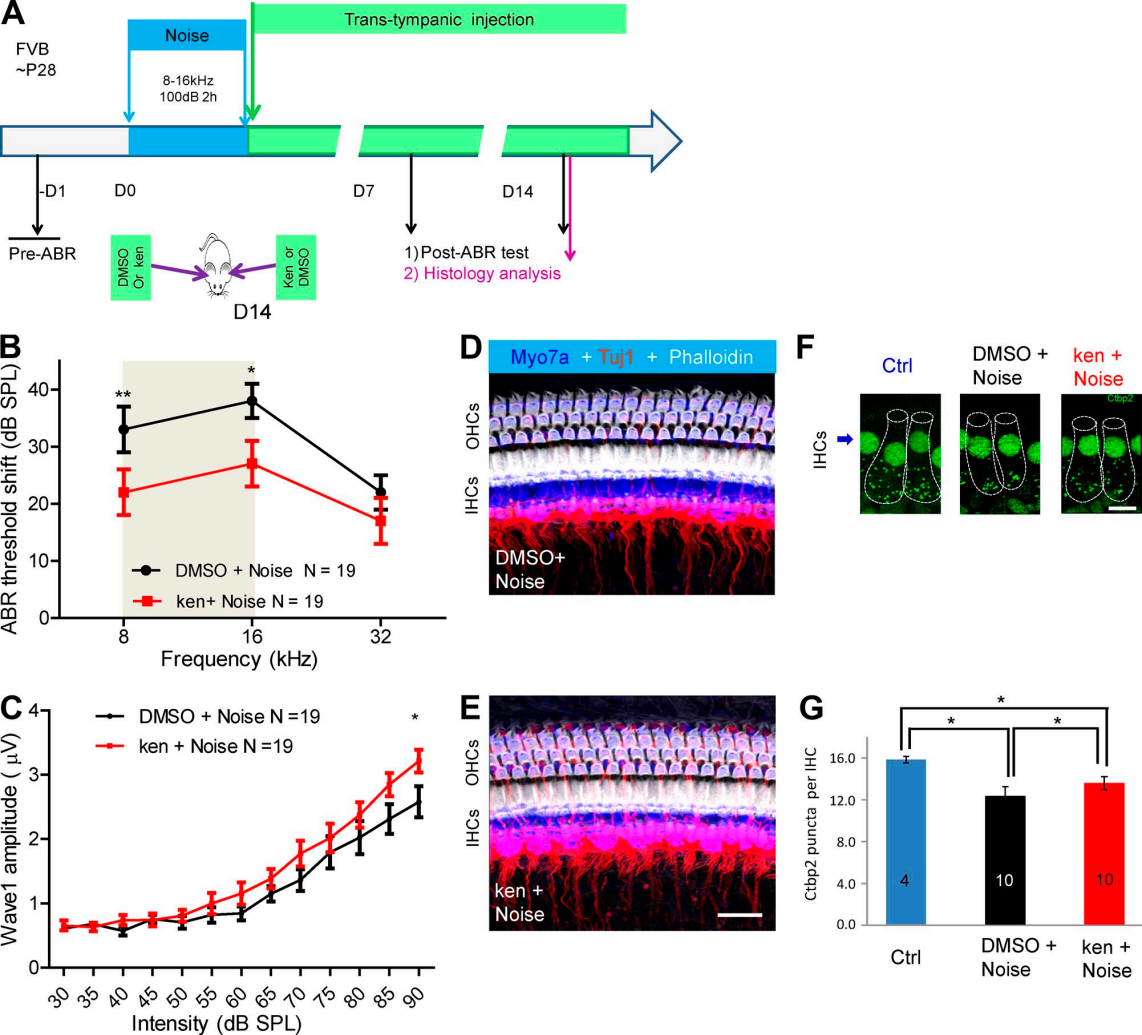
**Kenpaullone (ken) protects against noise-induced hearing loss in adult mice when delivered locally in vivo. (A)** Experimental design. Adult FVB mice at P28 were exposed to noise (8–16 kHz at 100 dB for 2 h). Immediately afterward, 5 µl ken (250 µM ken in 0.5% DMSO) was delivered to one ear of each mouse via trans-tympanic injection, and DMSO (0.5% in PBS) was delivered to the other ear, in a random order. ABR thresholds were recorded for each ear before and at D7 and D14 after noise exposure. Cochlear histology was examined at 14 d after exposure. All analysis was double blinded. **(B)** ABR threshold shifts of 19 mice at D14 in DMSO-treated (black) and ken-treated (red) ears. The gray area indicates the noise octave bandwidth between 8 and 16 kHz. **(C)** Amplitudes of ABR wave 1 at 16 kHz were significantly higher in ken-injected ears than in DMSO-injected ears. *, P < 0.05 at the 90-dB stimulus level by paired two-tailed Student’s *t* test. **(D and E)** Confocal images of similar cochlear regions (approximately the 16-kHz regions) of ken- and DMSO-treated ears triple stained with phalloidin, Tuj1, and myosin 7a (Myo7a). HCs and spiral ganglion neuron fiber innervation were intact in the 16-kHz region. Bar, 20 µM. **(F and G)** Comparison of the number of Ctbp2 puncta per IHC around the 16-kHz region in ken (ken+noise)- and DMSO (DMSO+noise)-treated ears with 100-dB noise damage and in untreated control ears (Ctrl). Cochleae analyzed were from 10 mice randomly chosen from the 19 mice analyzed in B and C; in each cochlea, 12–18 IHCs from 16-kHz regions were analyzed. Representative confocal images are shown in F, and puncta quantification of IHCs is demonstrated in G. IHCs are outlined with dashed lines in F. Bar, 10 µm. * P, < 0.05 and **, P < 0.01 by paired two-tailed Student’s *t* test. Results are presented as the mean ± SEM.

We originally hypothesized that protective compounds in our screen would be protective against both cisplatin- and noise-induced hearing loss because both conditions probably involve similar mechanisms of insult-induced cell death (Rybak and Ramkumar, 2007; Forge and Van De Water, 2008). To test for protection against noise by kenpaullone, we first developed the noise injury protocol in FVB adult mice (Fig. S4, A–C). Exposing mice to 100 dB of noise in the 8–16-kHz octave band for 2 h resulted in threshold elevations of 20–40 dB at D7 and D14 after exposure. Adult (P28) FVB mice were then subjected to this noise exposure condition and immediately afterwards were trans-tympanically injected with 250 µM kenpaullone (in 0.5% DMSO) in one ear and with carrier alone (0.5% DMSO) in the opposite ear (Fig. 4 A and Fig. S4, A–C).

As before, thresholds and wave 1 amplitudes of ABRs for both ears of each mouse were measured at D7 and D14. Kenpaullone-injected ears had significantly reduced threshold shifts when compared with DMSO-injected ears: the shifts were ∼12 dB at a frequency of 8 kHz at D7 and at frequencies of 8 and 16 kHz at D14 (Fig. 4 B and Fig. S4, D and E). Note that only the 8–16-kHz region, not the 32-kHz region, exhibited protection with kenpaullone, probably because of the 8–16-kHz octave band noise exposure (Maison et al., 2002). Similarly, at D14, the wave 1 amplitudes of ABRs at 16 kHz with various sound stimulus intensities were also higher in kenpaullone-treated ears than in DMSO-injected ears (Fig. 4 C). At D14, no HC loss or visible neuronal fiber damage was observed in noise-exposed ears, as determined by staining the cochleae for myosin 7a, Tuj1, and phalloidin (Fig. 4, D and E), which is consistent with previous studies (Maison et al., 2002; Wang et al., 2002). However, when we examined synaptic ribbons in the inner HCs (IHCs) where neuronal fibers formed synaptic contacts, there was less reduction in the presynaptic ribbon density at D14 after the acoustic damage in kenpaullone-treated, noise-damaged samples than in DMSO-treated, noise-damaged samples, as determined by quantifying the Ctbp2-positive puncta per IHC at the 16-kHz region (Fig. 4, F and G). Our measurements in DMSO-injected ears were consistent with results obtained in adult FVB mice exposed to similar noise (Maison et al., 2002). Interestingly, we found no significant protection by kenpaullone under similar conditions when mice were exposed to 106-, 112-, or 120-dB octave band noise (Fig. S4, F–H′). Together, the ABR threshold and wave 1 amplitude measurements and ribbon density analysis demonstrate that kenpaullone is protective against noise-induced hearing loss in mice in vivo.

### KO of *CDK2* confers resistance to cisplatin and noise injury

Pooled single-cell RNA sequencing showed that *CDK2* mRNA is highly expressed in adult OHCs and supporting cells (SCs; Fig. S5, A and B), which is consistent with a previous study (Scheffer et al., 2015). To test whether eliminating CDK2 would increase the resistance of inner ear cells to cisplatin, we obtained germline *CDK2* KO mice on a mixed background of CD1, FVB, 129, and C57BL/6 strains by crossing the original floxed allele with E2A-Cre. The KO mice were confirmed to lack CDK2 by immunoblot analysis (Fig. S5 C), and they were viable and exhibited normal hearing and normal morphology in the organ of Corti (Fig. S5, D–F).

**Figure 5.**
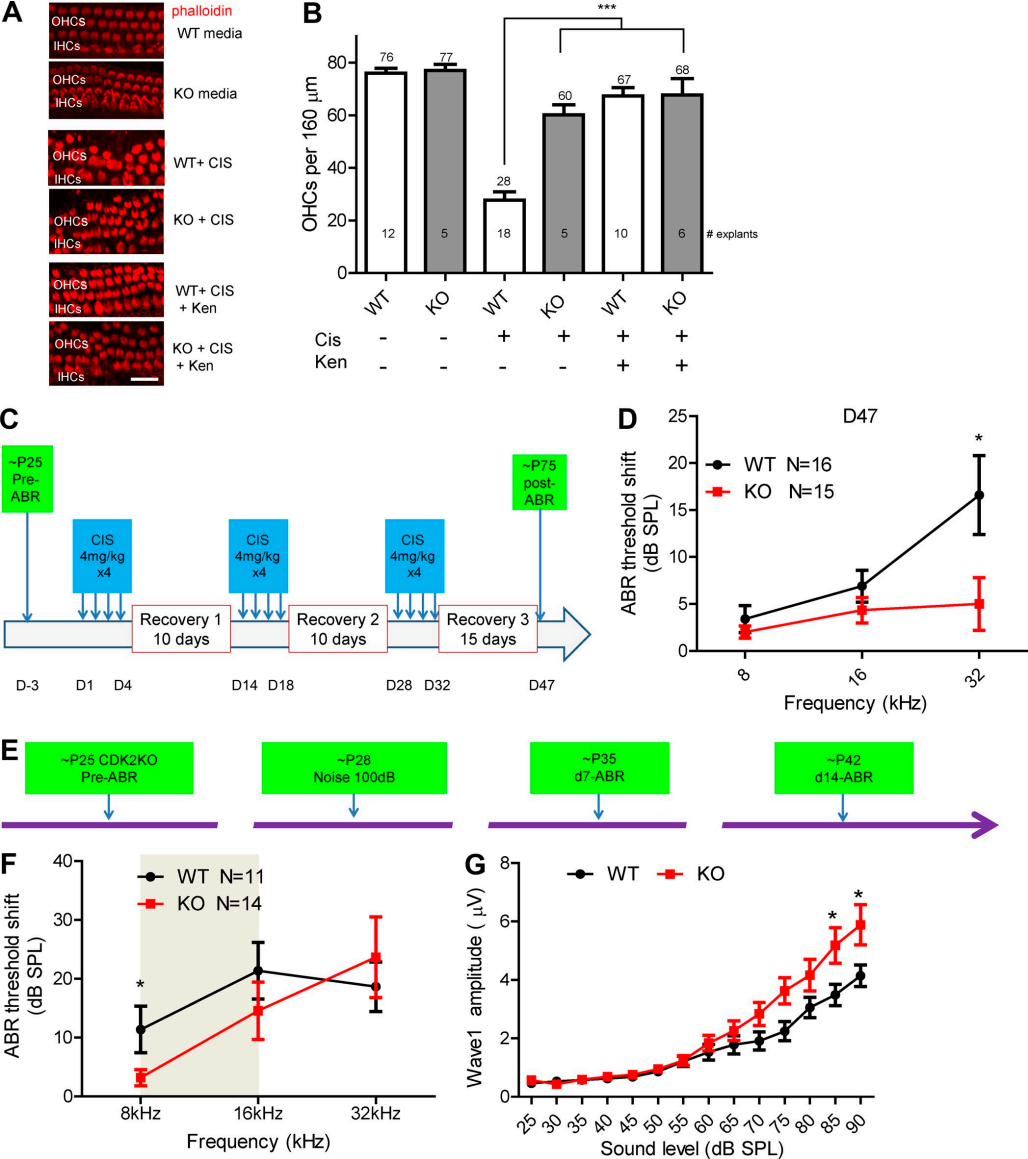
**Germline CDK2 KO cochlear explants exhibit resistance to cisplatin (CIS) treatment, and kenpaullone (ken) administration phenocopies CDK2 KO exhibit resistance to CIS ototoxicity.** All analysis here was double blinded. **(A)** Middle turn organs of Corti showing actin staining (with phalloidin) in CDK2 WT and KO cochleae with no treatment, with 50 µM CIS treatment, and with 50 µM CIS and 5 µM ken treatment. Bar, 20 µm. **(B)** The numbers of actin-labeled OHCs per 160 µm of the middle turn cochleae were counted. The numbers of explants tested in each condition are indicated in the bars. ***, P < 0.001 by one-way ANOVA with Bonferroni’s multiple comparison test. **(C)** Experimental design: *CDK2* KO and WT littermate mice were treated for 47 d with three rounds of treatment, each consisting of daily i.p. injections of 4 mg/kg cisplatin for 4 d followed by recovery for 10–15 d. **(D)** ABR threshold shifts at D47. *, P < 0.05 by unpaired two-tailed Student’s *t* test. Note that 11.6 dB was detected between WT and *CDK2* KO mice at 32 kHz. **(E)** Experimental design: CDK2 KO mice at P28 were exposed to noise (8–16 kHz at 100 dB for 2 h), and ABR thresholds were recorded before and at D7 and D14 after noise exposure. Cochlear histology was examined at D14. **(F)** Comparison of ABR threshold shifts at D14 in control mice (black) and CDK2 KO mice. The gray area indicates the noise octave bandwidth between 8 and 16 kHz. **(G)** Comparison of ABR wave 1 amplitudes at 8 kHz at D14 in WT and CDK2 KO mice. *, P < 0.05 by unpaired two-tailed Student’s *t* test. Error bars represent SEM.

Cochlear explants from the KO mice exhibited significantly enhanced resistance to cisplatin (Fig. 5, A and B). The CD1/FVB/129/C57BL/6 mixed background mice exhibited more cisplatin-induced HC loss than did the FVB background mice (Fig. 1, F–I). Thus, deleting *CDK2* enhanced cisplatin resistance in cochlear explants, phenocopying the protective effects of kenpaullone treatment in WT mouse cochlear explants. More importantly, adding kenpaullone to KO explants did not enhance resistance significantly (Fig. 5, A and B), suggesting that CDK2 is a major, if not the only, target of kenpaullone in the cochlea under these conditions and that this accounts for nearly all the protective activity of kenpaullone.

To test whether *CDK2* KO mice were protected against cisplatin ototoxicity in vivo, we adopted a protocol in which multiple low-dose cisplatin treatments were administered to mimic the cisplatin regimens used to treat patients with cancer (Roy et al., 2013). The protocol specified three rounds of daily treatment with 4 mg/kg cisplatin for 4 d followed by 10–15 d of recovery (Fig. 5 C). After 47 d of such treatments in each of three cohorts of adult WT and *CDK2* KO littermates, *CDK2* KO mice displayed complete protection against cisplatin ototoxicity at 32 kHz (representing a 11.6-db threshold shift, significantly lower than that in WT mice), whereas WT littermates exhibited 6.8- and 16.5-dB threshold shifts at 16 and 32 kHz (Fig. 5 D). These threshold shifts in WT mice were lower than those reported previously (Roy et al., 2013), probably reflecting the strain differences between the CBA/CaJ mice in the previous study and the CD1/FVB/129/C57BL/6 mice in this study. Regardless, the in vivo protection against cisplatin ototoxicity in *CDK2* KO mice provides strong evidence that *CDK2* KO phenocopies kenpaullone treatment and that kenpaullone is a potent therapeutic candidate for cisplatin ototoxicity in the clinic.

We further tested *CDK2* KO mice for protection against noise injury. When exposed to 100 dB of noise in the 8–16-kHz octave band for 2 h as before, KO mice displayed significantly smaller threshold shifts at 8 kHz at 7 and 14 d after exposure (Fig. 5, E and F; and Fig. S5, G–J), whereas at other frequencies no significant protection was observed. Similarly, wave 1 amplitudes of the ABRs were increased at D14, compared with those in WT littermates (Fig. 5 G). However, the synaptic puncta density of IHCs in KO mice at 8 kHz at D14 was not increased (Fig. S5 K). For the 106-dB noise level, no protection was observed at any frequency (Fig. S5, L and M). These results resemble those obtained with kenpaullone-treated FVB mice (Fig. 4), supporting the hypothesis that CDK2 is the target of kenpaullone.

### Kenpaullone directly inhibits CDK2 kinase activity

**Figure 6.**
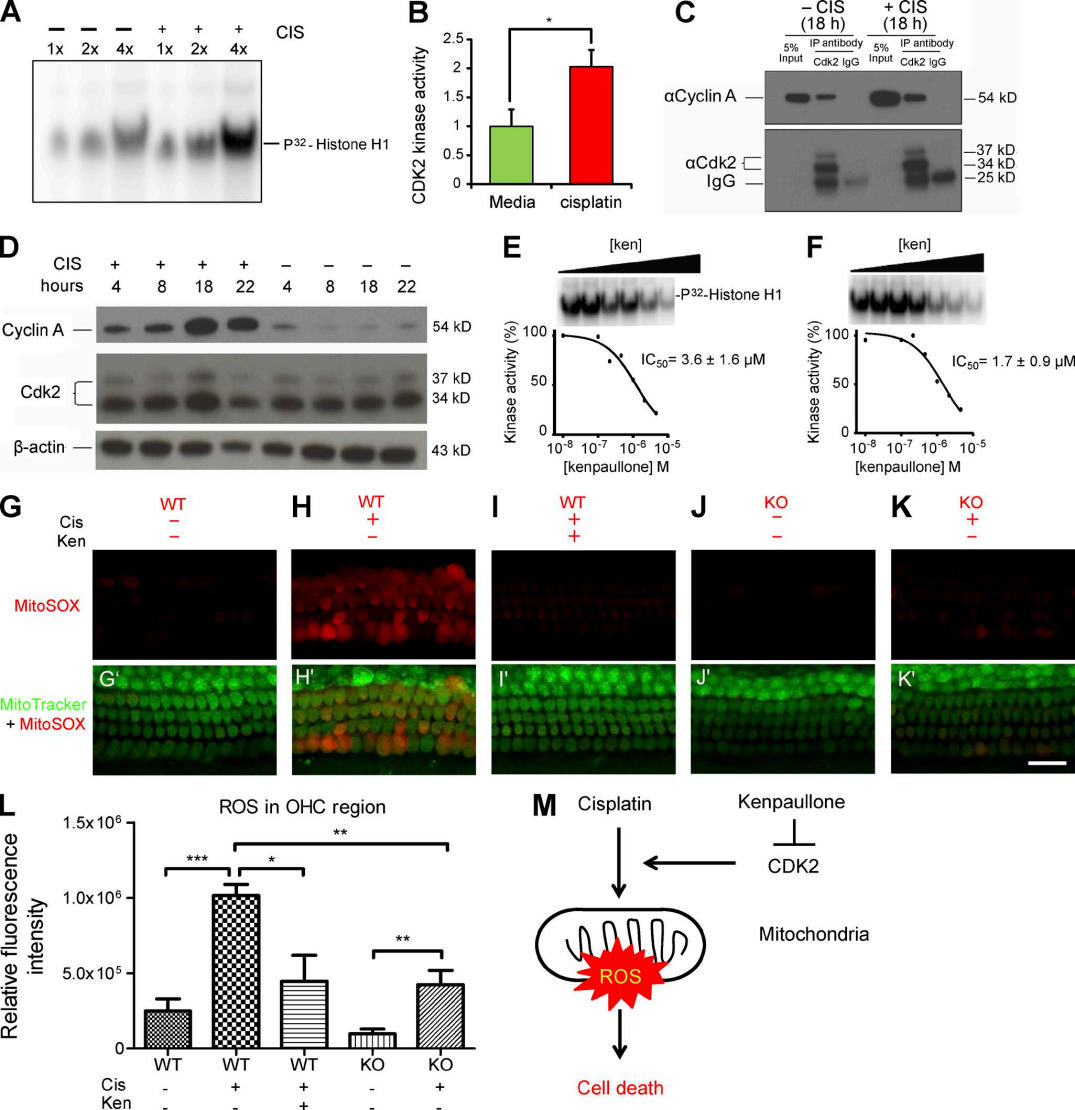
**CDK2 kinase activity is up-regulated after cisplatin (CIS) treatment; kenpaullone (ken) inhibits CDK2 kinase activity in HEI-OC1 cells and reduces CIS-induced, mitochondrial ROS production in cochlear explants. (A)** CDK2 kinase activity is up-regulated after CIS treatment in HEI-OC1 cells. Protein CDK2 was immunoprecipitated from HEI-OC1 cells treated with 50 µM CIS for 18 h with a CDK2-specific antibody (M2) and bound to Protein A agarose beads. The protein solution eluted from the beads was used in a kinase assay using [^32^P]histone H1 as the substrate, and different volumes (labeled 1×, 2×, and 4×) of the kinase reactions were loaded on an SDS-PAGE gel. **(B)** The CDK2 kinase activity reactions were performed in triplicate, and the intensities of the [^32^P]histone H1 bands for CIS-treated cells and untreated cells (green bar) were compared. **(C)** Cyclin A protein coimmunoprecipitated with CDK2 protein in HEI-OC1 cells treated and untreated with CIS. CDK2 was immunoprecipitated with a specific antibody (M2) from HEI-OC1 lysates treated or untreated with CIS for 18 h. Rabbit IgG was used as negative control. Equal amounts of immunoprecipitates and 5% of input were run on an SDS-PAGE gel; cyclin A and CDK2 were identified by Western blot analysis with specific antibodies. **(D)** Cyclin A is up-regulated in HEI-OC1 cells after CIS treatment. Cells were treated with 50 µM CIS or untreated and harvested at different time points. Cyclin A and CDK2 protein levels were compared by Western blot analysis with specific antibodies. β-Actin served as the loading control. **(E and F)** Ken inhibits CDK2–cyclin A kinase activity in HEI-OC1 cells untreated (E) and treated (F) with CIS. The dose response for ken inhibition of CDK2–cyclin A kinase was measured in the HEI-OC1 cells after 18 h in culture in medium as described in A, using [^32^P]histone H1 as the substrate. The IC_50_ was determined by curve fitting in triplicate experiments. The IC_50_ values are presented as the mean ± SD. **(G–K′)** Confocal images of the middle turns of organ of Corti explants labeled with MitoSOX red (G–K) or MitoTracker green and MitoSOX red (G'–K') under various conditions. CIS concentration was 150 µM. Bar, 20 µm. **(L)** Quantification of relative fluorescence intensities of MitoSOX ROS for each condition. CIS concentration was 150 µM. Data are presented as means ± SEM; *, P < 0.05; **, P < 0.01; and ***, P < 0.001. At least three different samples were analyzed for each condition. **(M)** Diagram of the mechanism by which ken and CDK2 KO protect against CIS-induced cell loss. CIS treatment induces mitochondrial ROS formation and thereby cell death. CDK2 KO or ken treatment decreases the ROS level induced by CIS treatment, further reducing cell loss.

To provide evidence that kenpaullone directly inhibited CDK2 kinase activity in cochlear cells, we immunoprecipitated CDK2 in HEI-OC1 cells 18 h after cisplatin treatment by using an antibody specific for CDK2. Using histone H1 as the substrate, we directly measured the CDK2 kinase activity and found that it was up-regulated twofold in cisplatin-treated cells, as compared with untreated cells (Fig. 6, A and B). Immunoprecipitation from HEI-OC1 cell lysates with the antibody to CDK2 brought down the regulatory subunit cyclin A with or without cisplatin treatment (Fig. 6 C). Consistently, immunoblot analysis of protein lysates from HEI-OC1 cells treated with cisplatin for 18 h showed dramatic up-regulation of the cyclin A protein that was already evident after 4 h of treatment and peaked at 18 h (Fig. 6 D). We saw no significant changes in the protein levels of CDK2, CDK4, or CDK6 (Fig. 6 D and Fig. S5 N). These results support the hypothesis that CDK2 and cyclin A interact in cochlear cells.

Next, we determined the IC_50_ of kenpaullone for the kinase activity of CDK2 immunoprecipitated from HEI-OC1 cells that were either untreated or treated with cisplatin for 18 h (Fig. 6, E and F). The IC_50_ values of 3.6 ± 1.6 µM and 1.7 ± 0.9 µM, respectively, were not statistically different from the IC_50_ of kenpaullone for cisplatin-induced caspase-3/7 activity in the HEI-OC1 cells (2.0 µM; Fig. 1 B). These results showed that kenpaullone directly inhibited CDK2 kinase activity as effectively as it inhibited cisplatin-induced caspase-3/7 activity in HEI-OC1 cells.

### CDK2 inhibition reduced mitochondrial production of ROS in cisplatin ototoxicity

To interrogate the downstream pathway of CDK2 inhibition in ototoxicity protection, we first analyzed changes in mitochondrial production of ROS in cisplatin-treated explants. Mitochondrial ROS production has been linked to ototoxicity and is upstream of caspase-3/7–induced cell death (Kim et al., 2010). We first quantified the relative fluorescence intensity of mitochondrial superoxide by using the indicator MitoSOX red. As expected, mitochondrial ROS production was cisplatin dose dependent (Fig. S5 V) and could be significantly reduced by the known antioxidant sodium thiosulfate (Fig. S5 W). Interestingly, cisplatin-induced mitochondrial production of ROS, as visualized with MitoSOX in HCs, was significantly reduced by CDK2 deficiency or kenpaullone treatment at 5 h after cisplatin treatment in cochlear explants (Fig. 6, G–L; and Fig. S5 X). These results are consistent with the cisplatin-induced HC loss seen in cochlear explants treated with kenpaullone or from CDK2 KO mice (Fig. 5, A and B), further supporting the hypothesis that CDK2 is a target of kenpaullone and suggesting that CDK2 inhibition attenuates cisplatin-induced mitochondrial ROS production and thereby caspase-3/7–mediated cell death (Fig. 6 M).

## Discussion

Given the alarming increase in noise-, antibiotic-, and cisplatin-induced and age-related hearing loss in the human population worldwide, the lack of FDA-approved drugs to treat or prevent this devastating disability is unacceptable. By using an unbiased, cell-based HTS, we have identified 10 top hits that appear to inhibit a wide range of biological processes converging on cisplatin-induced apoptotic cell death. Interestingly, 3 of these top 10 compounds (leflunomide, cobalamin, and olsalazine sodium; Table S1) are FDA approved for treating other diseases but have yet to be tested for treating inner ear diseases. In addition to the three CDK2 inhibitors (kenpaullone, olomoucine II, and CDK2 inhibitor II) characterized here, pelitinib (an irreversible inhibitor of epidermal growth factor receptor and an investigational new drug), antimycin A (an inhibitor of mitochondrial electron transport and inducer of apoptosis that has been tested in vivo in rodents), EHT1864 (an inhibitor of the Rac family of GTPases that has been tested in vivo in rodents), and d-ribofuranosylbenzimidazole (an inhibitor of CDK7/9 and RNA synthesis that has been tested in vitro) probably target diverse biological processes and warrant further characterization of their protective effects against ototoxicity in vivo. We originally hypothesized that because the diverse ototoxic insults (noise, cisplatin, antibiotics, and aging) shared similar apoptotic cell death pathways, our hit compounds would be effective against not just cisplatin-induced hearing loss, but also antibiotic-, noise-, and age-related hearing loss. Our extensive characterization of the protective effects of kenpaullone against both cisplatin and noise validates this hypothesis and suggests that other hit compounds reported here and their derivatives will also be effective in preventing hearing loss.

### Kenpaullone inhibits CDK2 to protect against cisplatin and noise injury

Surprisingly, among our top 10 hits, 3 (kenpaullone, olomoucine II, and CDK2 inhibitor II) are CDK2 inhibitors. Although each of these compounds generally inhibits multiple kinases, including CDK2 and GSK3-β (Zaharevitz et al., 1999; Yang et al., 2013), we have provided evidence that CDK2 is a major, direct molecular target of kenpaullone with respect to its protective effect against cisplatin-induced cochlear cell loss and noise-induced hearing loss. We therefore suggest that ubiquitously expressed CDK2 may play such a proapoptotic role in many postmitotic cell types to sense stress levels, similar to the role of p53. Because kenpaullone was a candidate therapeutic for amyotrophic lateral sclerosis and cardiac parasympathetic dysfunction (Yang et al., 2013; Zhang et al., 2014), our findings suggest that CDK2 might also contribute to the protective effects of kenpaullone against amyotrophic lateral sclerosis and cardiac parasympathetic dysfunction.

### Protective mechanisms of CDK2 inhibition in postmitotic cells

In this paper, we provide mechanistic evidence of how kenpaullone treatment or CDK2 inhibition protects against cisplatin-induced cell death in postmitotic cochlear cells (summarized in Fig. 6 M). After cisplatin induction, CDK2 kinase activity is activated, mostly by the up-regulation of its cofactor cyclin A, but its inhibition by kenpaullone or CDK2 KO results in reduced mitochondrial ROS production and reduced caspase-3/7–mediated activation of apoptosis. Our results are consistent with previous studies that CDK2 activation is required for the disruption of mitochondrial permeability in apoptosis by various insults in the kidney, thymus, and liver (Jin et al., 2003; Granés et al., 2004; Guo et al., 2011; Megyesi et al., 2016; Seng et al., 2016). An unpublished study has reported that the inhibition of CDK2 is also protective against antibiotic ototoxicity in the cochlea (Tao, 2014), further supporting our conclusion that CDK2 inhibition protects against hearing loss caused by diverse ototoxic insults.

Traditionally, cisplatin has been considered a DNA-chelating agent, causing DNA damage that leads to repair and cell cycle reentry or apoptosis in proliferating cells (Fang et al., 2016). However, we detected no significant staining for γ-H2AX in cochleae (Fig. S5, O–U′), which is evidence that ototoxic insults exert nonnuclear (cytoplasmic or mitochondrial) effects in postmitotic cochlear cells (Galluzzi et al., 2012). However, it remains unclear how cisplatin or noise injury induces the up-regulation of the CDK2 cofactor (i.e., cyclin A) and how the increased CDK2 kinase activity leads to enhanced mitochondrial ROS production. The simplest scenario is that cisplatin-activated CDK2 kinase directly phosphorylates molecular targets in mitochondria, leading to ROS production, caspase 3 activation, and ultimately cell death. Because CDK2 is expressed in multiple cell types in the cochlea (Fig. S5, A and B), kenpaullone might act through a cell-nonautonomous mechanism that protects globally against cochlear insults. In addition, kenpaullone may exert its effect not only by protecting HCs (Fig. 3) but also by protecting stria vascularis cells and thus attenuating the endocochlear potential in cisplatin-treated cells. Recent publications show that cisplatin is retained in the mouse and human stria vascularis cells (Rybak et al., 2007; Breglio et al., 2017). Alternatively, kenpaullone may have an effect on fibrocyte turnover and proliferation in the spiral ligament after cisplatin and/or acoustic trauma (Roberson and Rubel, 1994; Yamashita et al., 1999; Hirose and Liberman, 2003; Lang et al., 2003). These hypotheses remain to be tested in the future.

Our results demonstrate that CDK2 inhibition protects against hearing loss caused by diverse ototoxic insults. However, the protective mechanisms underlying CDK2 inhibition appear to differ for noise- and cisplatin-induced injury. Unlike cisplatin ototoxicity, exposure to 100-dB noise causes no HC loss but does result in ribbon synapse reduction and permanent hearing loss. It remains unclear why kenpaullone treatment or CDK2 KO conferred protection against noise exposure at 100 dB but not at 106 dB or higher under our experimental conditions. It is therefore possible that CDK2 inhibition is more effective at preventing hidden hearing loss, a condition in which low levels of noise (≤94 dB) cause no transient or permanent hearing loss but do result in obvious synaptic changes (Kujawa and Liberman, 2009). This hypothesis warrants further investigation in our mouse models. Given that 100-dB noise is in the range of noise insults commonly experienced by people in our society, kenpaullone could have significant clinical application in treating noise-induced hearing loss.

### Therapeutic potential of locally delivered kenpaullone

We have focused on characterizing kenpaullone to provide a proof of concept for its potential development as a candidate therapeutic to prevent hearing loss. Using identical conditions in cochlear explant cultures treated with cisplatin, we have performed a side by side direct comparison of kenpaullone and four benchmark compounds that are currently in clinical trials: d-methionine, ebselen, sodium thiosulfate, and dexamethasone. Kenpaullone not only provided complete OHC protection but also exhibited higher potency and/or a better therapeutic index than the four benchmark compounds. Moreover, having been originally developed as anticancer agents that do not interfere with the tumor-killing ability of cisplatin, CDK2 inhibitors are superior to many other benchmark compounds in preclinical and clinical trials against ototoxicity (Fig. S2). Local delivery of drugs into the middle ear via the trans-tympanic membrane has been widely used (El Kechai et al., 2015; Lambert et al., 2016), and kenpaullone is particularly suitable for in vivo local delivery because of its high potency, minimal toxicity, and low aqueous solubility. Given our success in demonstrating that the robust protection conferred by one-time locally delivered kenpaullone and CDK2 inhibition in various mouse and rat models reached as high as 40 dB at multiple frequencies, our discovery of CDK2 inhibitors that act as otoprotectants will probably transform the clinical prevention and treatment of cisplatin ototoxicity and noise-induced hearing loss in patients. Modifications of the treatment regimens, additional optimization of the delivery methods via the use of hydrogels, and structural modifications of the compounds via medicinal chemistry could ensure even better results with CDK2 inhibitors in treating hearing loss in humans.

## Materials and methods

### Mouse models

FVB breeding mice were purchased from the Jackson Laboratory and bred in the St. Jude animal facility. FVB mice were used for the noise damage and cisplatin treatment experiments. C57BL/6/129 mixed strains were used for the cochlear explant experiments to test the top hits. CDK2-floxed mice obtained from European Mouse Mutant Archive (05931) contained the CD1 outbred strain background, in addition to the FVB and 129 inbred strain backgrounds. The genotypes of CDK2 alleles were determined based on a previous study (Ortega et al., 2003). The germline CDK2 KO mice were created by crossing CDK2-floxed/floxed mice with EIIA-Cre on C57BL/6/129 mixed backgrounds, from which offspring possessing the *CDK2* deletion and lacking Cre were intercrossed to obtain homozygous CDK2 germline KO mice. All animal experiments were approved by the Institutional Animal Care and Use Committee of St. Jude Children’s Research Hospital.

### Tumor cell lines

The three mouse group 3 MB tumorsphere lines were described previously (Morfouace et al., 2014). The two human NB cell lines, IMR32 and SH-SYSH (with and without *N-MYC* amplification, respectively), were grown as described previously (Barbieri et al., 2006; Altun et al., 2010).

### Cell-based primary HTS

For this screen, caspase-3 cleavage was chosen as the endpoint indicating cisplatin-induced cell death, as it allowed the inhibition of cell death to be monitored at the level of any intracellular molecular target upstream of caspase-3 cleavage in the HEI-OC1 cell line (Kalinec et al., 2003; Kim et al., 2010). Pifithrin-α was chosen as a reference compound for the screen, as it provided good protection against cisplatin ototoxicity by inhibiting caspase-3 cleavage with an IC_50_ of 17 µM (Fig. S1 D; Zhang et al., 2003; Ding et al., 2012; Teitz et al., 2016). This screen has been described in detail elsewhere (Teitz et al., 2016). Assays were first optimized at the bench. Optimized aspects included the number of cells to be plated (1,600 cells/well), the cisplatin concentration (50 µM, based on the dose–response curve), the incubation time (22 h at 33°C in 10% CO_2_), and the concentration of pifithrin-α (17 µM). The Caspase-Glo 3/7 assay (Promega), which enables the measurement of light emitted as the result of caspase cleavage and is suitable for HTS, was used. The linearity of the Caspase-Glo 3/7 assay was validated, and it was verified that 0.5% DMSO had no effect on the cell death kinetics (Hall et al., 2014). Compound toxicity was tested by cell viability assay CellTiter-Glo (Promega) with compound alone. The Caspase-Glo 3/7 assay was then tested for reproducibility in the St. Jude robotic systems in 384-well plates (white, solid-bottom, tissue culture–treated, polystyrene plates; Corning). By using a pin tool, test compounds dissolved in DMSO were added to the screen to a final concentration of 8 µM, and cisplatin solution in PBS was added 2 min later to each well to a final concentration of 50 µM (in <0.5% DMSO; Fig. S1 A). The cells were incubated with the test compounds and cisplatin for 22 h at 33°C in 10% CO_2_, with the medium being as previously described (Kalinec et al., 2003); no γ IFN was added to slow the cells’ growth rate and to mimic as closely as possible the conditions of postmitotic, nondividing sensory epithelial (organ of Corti) cells. The 384-well plates were shaken and centrifuged after compound addition to enhance the reproducibility of the assay. Pifithrin-α was added to each plate as a screening quality control. Among the top hits were several previously characterized otoprotective agents, such as zVAD-FMK (a known general, irreversible protein inhibitor of caspases [Teitz et al., 2016]), ebselen (currently being tested against noise injury in a clinical trial; https://clinicaltrials.gov/show/NCT01444846, accessed February 21, 2018), olsalazine sodium (a derivative of salicylate; Fig. S1 F), and cyanocobalamin (vitamin B12; Fig. S1 G).

### Cochlear explants

P3 C57B6/129SvEv mouse cochleae were dissected and maintained in culture with the aid of Matrigel as previously described (Driver and Kelley, 2010). After cells had been in culture for 1 d, growth medium DMEM (12430-054; GIBCO Life Technologies, with 1% FBS [16000-044; GIBCO Life Technologies] and 50 µg/ml ampicillin) with or without the test compound was added for preincubation for 1 h at 37°C in 5% CO_2_, and this was followed by incubation with 50 µM cisplatin (479306; Sigma-Aldrich) with or without the test compound in growth medium for 24 h at 37°C. A cisplatin concentration of 50 µM was chosen because the explant assay consistently showed that ∼40% of OHCs in the mouse cochlea died at this concentration after 24 h (Fig. 1, E–K). OHCs are the first cells to be damaged by cisplatin and noise both ex vivo and in vivo, whereas IHCs are injured only at higher concentrations of cisplatin or higher levels of noise (Zhang et al., 2003; Oishi and Schacht, 2011). Cochleae were fixed with 4% PFA and stained for actin with Alexa Fluor 568 phalloidin to determine the viability of the HCs, which was also assayed by DAPI staining, FM1-43 dye uptake, and immunohistochemical staining for known HC markers (parvalbumin and myosin 7a). Cochleae were imaged by confocal microscopy, two 160-µm regions from middle turns were photographed, and the number of intact HCs was counted. At least three independent cochleae were tested for each experimental condition.

For comparisons between kenpaullone and four benchmark compounds in explants, we used similar procedures as above with the following modifications: (a) we used filters (Millicell, PICM03050; Millipore) instead of Matrigel in 6-well culture plates with 1 ml medium solution both inside and outside the filter, (b) we used the P3 FVB mouse strain, and (c) we used a cisplatin dose of 150 µM that consistently caused the loss of ∼40% of OHCs within 24 h, based on the dose responses of cisplatin at 50, 100, 150, and 200 µM.

To further characterize the time course of cisplatin and kenpaullone cotreatment, we treated explants with 150 µM cisplatin for 48 h and found that, in contrast to 24-h cisplatin incubation, all OHCs died in P3 FVB mouse explants (*n* = 4). The cotreatment with cisplatin and kenpaullone (100 nM to 30 µM) for 48 h did not show any protection (*n* = 7), whereas this cotreatment showed complete protection when explants were incubated for 24 h (Fig. 1, E–I; and Fig. S2, A and B). For cisplatin washout experiments, when cisplatin was removed after incubation for 90 min (an estimated time that cisplatin stays in the inner ear in vivo after i.p. injection; Hellberg et al., 2009), we found only 11% OHC death (*n* = 3) as compared with 40% OHC death with a 24-h 150 µM cisplatin incubation as we described in Fig. 1 [E–I] and Fig. S2 [A and B]. When 5 µM kenpaullone was added for the entire 24 h, full protection against cisplatin was observed (*n* = 4).

### Zebrafish lateral lines

Zebrafish *AB strains were provided by W. Clements (St. Jude Children’s Research Hospital, Memphis, TN). Experiments were performed in 24-well plates with five fish per well in volumes of 1–2 ml. Fish were incubated with each compound (Table S1) and stained with 0.005% DASPEI vital dye for 15 min. After two washes with egg water (0.03% Instant Ocean), fish were visualized in an epifluorescence microscope. 10 specific neuromasts in each fish were scored based on their staining intensity on a scale ranging from 0 (no labeling) to 2 (high intensity), as described previously (Owens et al., 2009; Buck et al., 2012). Results were plotted as the DASPEI intensity score in the cisplatin-treated fish relative to that in the untreated fish (Fig. 2, A–E). There are many possible reasons why 9 of the 10 top hit compounds, including olomoucine II and CDK2 inhibitor II, did not confer protection under our tested assay conditions; for example, the cisplatin damage to the neuromasts might have been too severe, the uptake of compounds by the neuromasts might have been limited, zebrafish neuromasts have different anatomical structures (i.e., separation of fluid spaces), and neuromasts might have different protective intracellular mechanisms to those of mammalian neuromasts (Owens et al., 2009; Ou et al., 2012; Vlasits et al., 2012; Thomas et al., 2013).

### Trans-tympanic injection in adult mice and rats

Adult animals were anesthetized by i.p. injection of avertin (mice) or inhalation of isoflurane (rats). Their body temperature was maintained with a heating pad during the surgical procedure. Lubricant eye ointment was applied to prevent corneal ulcers, as the blinking reflex disappears during surgery. The tympanic membrane was visualized with a surgical stereomicroscope. By using a 33-G cannula, 5 µl (for mice) or 30 µl (for rats) of compound in 0.5% DMSO in PBS or alternatively 0.5% DMSO in PBS alone was gently injected through the tympanic membrane, after which the surgical stereomicroscope was used to confirm that the solution was in the middle ear cavity. Either ear of each mouse might have been injected with either compound or control in a random order. For rats, only the left ear was injected. After each injection, the animals were placed in their cage and kept on the heating pad for a further 30 min with their injected ears upward. Injected animals were allowed to recover on the heating pad before being returned to the animal housing facility. To test the efficacy of trans-tympanic delivery of drugs, we first injected a dye (FM1-43) trans-tympanically in mice and found that the uptake of the dye by HCs and the injection itself had no obvious adverse effects on hearing after 7 d (Fig. S3, A–E). The experiments were performed in a double-blind manner, and the order in which the ears were injected was switched between mice to minimize bias.

### Cisplatin treatment in mice

10 mg cisplatin (479306; Sigma-Aldrich) powder was dissolved in 10 ml sterile saline (0.9% NaCl) at 37°C for 40–60 min. To determine the ototoxic effects of cisplatin in FVB mice, cisplatin (1 mg/ml stock in saline) at different doses (15, 20, 25, and 30 mg/kg body weight) was administered by i.p. injection in one to three mice at each dose in three independent cohorts, and ABR threshold shifts were measured at 14 d after cisplatin injections. 1 d before cisplatin injection, the mice received 1 ml saline by subcutaneous injection. To ameliorate dehydration after cisplatin injection, 1 ml of warm saline was injected twice per day for at least 7 d until body weight started to recover. The cages of cisplatin-treated mice were placed on the heating pad for at least 7 d. Mush food was daily given for at least 7 d. Cisplatin doses < 30 mg/kg did not cause significant ototoxicity at D14 after cisplatin injection. Hearing loss was detected with a dose of 30 mg/kg, whereas several mice that received that dose reached the humane endpoint before undergoing hearing tests. To test the protective effect of kenpaullone, a total of 20 mice were divided into four cohorts of five mice each. Three mice died under anesthesia during trans-tympanic injection procedures, and six mice reached the humane endpoint before the ABRs were measured at 7 d after injection. For multiple low-dose cisplatin treatments, littermate control and CDK2 KO mice received three cycles cisplatin treatment (4 mg/kg body weight × 4) followed by 10–15 d of recovery as previously described (Roy et al., 2013). A total of 31 mice in four cohorts were tested.

### Kenpaullone injection in cisplatin-treated adult rats

Wistar rats were purchased from Charles River Laboratory (stock 003) and were housed with two rats per cage and acclimated for more than 1 wk. First, to determine the nontoxic maximal dose of kenpaullone in rats, we tested doses of 0, 38.5, 77.5, 155, and 310 µM kenpaullone alone (in 0.5% DMSO in PBS) by trans-tympanic injection in two to four rats of both sexes and measured the ABR threshold shifts at 4, 8, 16, and 22 kHz at 4–7 d after injection. A dose of 310 µM kenpaullone alone induced no significant ototoxicity in rats, even after 30 d (Fig. 3 F). We first tested the ABR thresholds of each rat at 4, 8, 16, and 22 kHz while they were anesthetized with an i.p. injection of 1.5 ml/kg rat ketamine/xylazine (RKX) cocktail consisting of 86 mg/ml ketamine and 2.76 mg/ml xylazine (Campbell et al., 1996). One rat with high thresholds >40 dB at multiple frequencies was excluded. For 13 mg/kg cisplatin treatment, we tested 12 rats (body weight 306–354 g) in two cohorts; for 16 mg/kg cisplatin treatment, we tested 24 rats (body weight 226–371 g) in four cohorts. At least 3 d after precisplatin ABR tests, each rat was anesthetized with 4% isoflurane in oxygen (2 liter/min) for induction in a chamber and maintained with 2% isoflurane in oxygen (2 liter/min), whereas 310 µM kenpaullone or carrier (0.5% DMSO in PBS) was injected trans-tympanically into the left ear. 1 h later, 13 or 16 mg/kg freshly prepared cisplatin (1 mg/ml) was administered i.p. at a rate of 0.22 ml/min with an infusion pump (Kent Scientific). To lessen dehydration and kidney toxicity, we injected each rat with 10 ml saline subcutaneously twice daily from 1 d before the cisplatin treatment until a humane endpoint was reached. A mean bodyweight loss of 16.7% ± 0.9% (mean ± SEM) at postcisplatin treatment D4. At D4, we measured the ABR thresholds of each rat while it was anesthetized with RKX as described above. For the 13 mg/kg cohorts, one rat died during cisplatin injection under anesthesia. For the 16 mg/kg cisplatin cohorts, three rats that reached humane endpoints before the D4 ABR tests, and three rats that did not recover from anesthesia during the D4 ABR tests were excluded. ABR thresholds were evaluated by two independent investigators who were blinded to the kenpaullone or PBS/DMSO injection status of each rat until all the data were collected.

### ABR threshold and wave 1 amplitude measurements

The closed-field (kenpaullone or DMSO injection in mice) or open-field (CDK2 KO mice and rats) ABR was measured for each ear as described previously but with minor modifications (Yamashita et al., 2012; Liu et al., 2014). In brief, ABR waveforms were recorded in a sound booth (Industrial Acoustic Company) by using subdermal needles positioned in the skull, below the pinna, and at the base of the tail, and the responses were fed into a low-impedance Medusa digital biological amplifier system (RA4L; TDT; 20-dB gain). At each frequency, the stimulus intensity was reduced from 90 to 0 dB in 5-dB steps to determine the threshold decibel sound pressure level (SPL) when the electrical response was just above the noise floor. ABR waveforms were averaged in response to 500 tone bursts. The recorded signals were filtered by a band-pass filter from 300 Hz to 3 kHz. Individual ABR wave 1 amplitudes were measured as the difference between the positive peak and the following negative trough.

### Noise exposure in mice

We exposed the mice to noise before performing the trans-tympanic injections to ensure that the noise exposure was as specified and was not mitigated by procedure-induced temporal hearing loss (Fig. 4, A–C), to ensure that the damage was similar to that recorded in previous studies (Maison et al., 2002; Ho et al., 2014) on noise damage in FVB mice, and to mimic certain clinical conditions in which protective drugs might be delivered after exposure. Because of the mixed-strain CD1/FVB/129/C57BL/6 background, CDK2 KO and WT littermate mice exhibited greater variability in their ABR thresholds than did FVB or 129/C57BL/6 mice, especially at 32 kHz (Fig. 5 F and Fig. S5, G–I).

Mice were placed in individual cages in a custom-made acrylic chamber. The sound stimulus was produced by System RZ6 (TDT) equipment and amplified using a 75-A power amplifier (Crown). Sound was delivered to the acrylic chamber via a speaker horn (JBL). The SPL was calibrated with a 1/4-inch free-field microphone (PCB). Before experimental noise exposure, four quadrants of the cage inside the chamber were sampled with the 1/4-inch microphone to ensure that the SPL varied by <0.5 dB across the measured positions. Adult mice were then exposed to 2 h of octave band noise (8–16 kHz) at SPLs between 100 and 120 dB.

### Tissue preparation, immunofluorescence, and quantification analysis

Tissues from adult mice were prepared and examined as described previously (Wu et al., 2004; Yamashita et al., 2012). All images were acquired with a confocal microscope (LSM 700 or 710; Zeiss). The cells were stained with phalloidin (A12379 or A12380; Invitrogen) or with antibodies for other markers. The following primary antibodies were used: anti–myosin 7a (1:200; 25-6790; Proteus Bioscience), antiparvalbumin (1:2,000, P3088; Sigma-Aldrich), anti-Ctbp2 (1:500; 612044; BD Transduction), and anti-Tuj1 (1:1,000; MMS-435P; Covance). All secondary antibodies were purchased from Invitrogen and used at a 1:1,000 dilution. Hoechst (1:1,000; H3570; Invitrogen) or ProLong gold antifade reagent with DAPI (P36941; Invitrogen) was used to counterstain nuclei.

### Ctbp2 staining and quantification

Organs of Corti were costained with anti-Ctbp2 and anti–myosin 7a antibodies. Confocal imaging was performed using a Zeiss 710 or 700 scanning confocal microscope with a 63× objective. Visualization and projections were performed using ZEN 2009 or ZEN 2012 software (Zeiss). Ctbp2 puncta were visualized and counted on the reconstructed three-dimensional images by using the ZEN 2009 or ZEN 2012 three-dimensional construct function as previously reported (Liu et al., 2014). 4 cochleae from 2 FVB mice and 20 cochleae from 10 FVB mice were randomly chosen from 19 that had noise damage, and kenpaullone or DMSO injections were used to quantify the Ctbp2 puncta. Images were acquired around 16-kHz cochlear regions, and each image included 12–18 IHCs that were counted.

### Immunoblotting and immunoprecipitation

Total lysates of cells or dissected organs of Corti were prepared in lysis buffer (9803; Cell Signaling Technology) after adding protease and phosphatase inhibitors (Roche). Organs of Corti were dissected on ice in cold PBS containing the aforementioned inhibitors. Total cell lysates were sonicated (20% amplitude, four times for 5 s each at 4°C), and organ of Corti tissues were dissociated in 30–40-µl volumes with glass beads at 4°C by using a blender (24 Gold; Bullet) at speed 6 for four times for 30 s. After sonication (for cells) or dissociation in the blender (for organ of Corti tissues), the lysates were centrifuged for 10 min at 15,000 *g* at 4°C, and the supernatants were collected. Protein concentrations in protein solutions were determined with the BCA protein assay kit (23235; Thermo Scientific). 10 µg of total cell lysates or the equivalent of four to six cochleae protein lysates was loaded on 5–20% SDS-PAGE gels. For immunoprecipitation, HEI-OC1 cell lysates (250 µg total protein) were prepared in the presence of protease and phosphatase inhibitors and immunoprecipitated with 10 µl (2 µg) anti-CDK2 antibody (M2, rabbit polyclonal IgG antibody; Santa Cruz Biotechnology) and 20 µl of a 50% slurry of Protein A agarose beads (Cell Signaling Technology). An equal amount of rabbit IgG (2 µg) was used in parallel immunoprecipitation reactions with 250 µg total protein as a negative control. After 1 h preclearing with the Protein A agarose beads at 4°C, anti-CDK2 M2 antibody was added and incubated overnight at 4°C with the protein lysates. 5% of the precleared protein solutions (input) and equal portions of the immunoprecipitates were separated on 5–20% SDS-PAGE gels and immunoblotted with anti–cyclin A monoclonal antibody (E23.1) and anti-CDK2 antibody (M2). The following antibodies were used for immunoblot analysis or immunoprecipitation: anti-CDK2 (M2; SC-163; Santa Cruz Biotechnology), anti–cyclin A (H-432; SC-751; Santa Cruz Biotechnology), anti–cyclin A monoclonal antibody (E23.1; Thermo Scientific), anti-CDK4 (C-22; SC-260; Santa Cruz Biotechnology), anti-CDK6 (3136; Cell Signaling Technology), and anti–β-actin (C4; SC-47778). The antibodies were used at dilutions ranging from 1:500 to 1:1,000.

### CDK2–Cyclin A kinase activity assay

HEI-OC1 cell lysates (250 µg total protein) were prepared in the presence of protease and phosphatase inhibitors and immunoprecipitated with 10 µl anti-CDK2 antibody (M2; Santa Cruz Biotechnology) and 20 µl of a 50% slurry of Protein A agarose beads (Cell Signaling Technology). After mixing and washing the beads, the eluted protein solution was used in a kinase assay containing ATP, 15 µM histone H1 (EMD Millipore), and 6 µCi γ-[^32^P]ATP (PerkinElmer; Giono and Manfredi, 2007; Iconaru et al., 2015). Each reaction mixture had a total volume of 20 µl and was incubated for 35 min at 30°C. The sample buffer contained 20 mM Hepes, pH 7.3, 25 mM sodium β-glycerolphosphate, 15 mM MgCl_2_, 16 mM EGTA, 0.5 mM Na_3_VO_4_, and 10 mM DTT. The reactions were quenched by adding 5 µl SDS gel–loading buffer, and then 10-µl samples of the reaction mixtures were analyzed by SDS-PAGE. The gels were dried at 70°C under vacuum, and a phosphorimager (GE Healthcare) was used to quantify the [^32^P]histone H1 bands. IC_50_ values were determined by curve fitting using KaleidaGraph software. Experiments were performed in triplicate, and the mean IC_50_ and standard deviations of the mean values are reported. We used purified CDK2–cyclin A complex to quantify the IC_50_ of kenpaullone for CDK2 and found it be 0.46 µM, which is close to the IC_50_ of 0.68 µM reported for CDK2 in the literature (Zaharevitz et al., 1999).

### Analysis of ROS production in cochlear HCs

To measure the ROS changes, the explants of organs of Corti from WT and CDK2 KO mice were maintained in culture on filters (cell culture inserts, Millicell-CM; PICM03050; Merck Millipore) at 37°C in 5% CO_2_ overnight. The next day, these explants were incubated in Millicell filters with 50 µM cisplatin alone or were preincubated with 5 µM kenpaullone for 1 h, followed by incubation with 5 µM kenpaullone and 50 µM cisplatin for 5 h. After a 5-h incubation, the explants were washed twice with DMEM only, incubated with 500 nM MitoTracker green (M7514; Thermo Scientific) and 5 µM MitoSOX red (M36008; Thermo Scientific) in DMEM at 37°C in 5% CO_2_ for 1 h, then washed with DMEM, and placed on glass slides. Fluorescence images were immediately obtained using a confocal microscope (Zeiss 700). ImageJ software (National Institutes of Health) was used to quantify the relative fluorescence intensity in accordance with the instructions on measuring cell fluorescence using ImageJ (Burgess et al., 2010; McCloy et al., 2014). All confocal images were acquired with the equipment in an identical configuration (i.e., laser intensity, pin hole, gain, and z stake interval). The images were projected and imported into ImageJ. The total immunofluorescence of MitoSOX in the OHC regions was measured for each treatment condition. The mean intensity of the background fluorescence near the OHCs was also measured to calculate the background fluorescence in the OHC region. The relative fluorescence was quantified by subtracting the background fluorescence for the same area of the OHC region from the total fluorescence intensity in the OHC region. MitoSOX fluorescence intensity in the IHC regions was determined similarly. Each condition was replicated in at least three different cochlear explants. Cisplatin dose responses and sodium thiosulfate control experiments were performed in FVB explants, in which 150 µM cisplatin caused the loss of ∼40% of OHCs, which was similar to the loss of OHCs caused by 50 µM cisplatin in C57B6/129 explants.

### Statistical analysis

Statistics was performed by using Prism (GraphPad Software). We used one-way ANOVAs with Bonferroni post hoc test for mean difference or a two sample Student’s *t* test if only two conditions were compared.

### Online supplemental material

Fig. S1 shows the outline of the HTS of bioactive compounds, the dose–responses of the top seven hits in the screen in addition to the three CDK2 inhibitors, and cisplatin dose–response curves in the mouse cochlear explants. Fig. S2 compares kenpaullone with four known benchmark protective compounds in cochlear explants and tests the potential interference of the top 10 hits with cisplatin’s killing ability in five tumor lines (MB and NB). Fig. S3 describes the local delivery of kenpaullone in cisplatin-treated mice and rats. Fig. S4 describes the local delivery of kenpaullone in noise-exposed mice. Fig. S5 describes CDK2 expression in cochleae and CDK2 KO mice in cisplatin- and noise-induced hearing loss; no expression changes were detected in Cdk4, Cdk6 protein levels or in γ H2AX protein staining after cisplatin treatment. Mitochondrial superoxide production as measured by the indicator MitoSOX red was cisplatin dose dependent and was significantly reduced by the known antioxidant sodium thiosulfate. Table S1 shows the top hits for ototoxicity protection characterized in the study.

## Supplementary Material

Supplemental Materials (PDF)
