# Supplementary material for: CDK2 inhibitors as candidate therapeutics for cisplatin- and noise-induced hearing loss
Source: J Exp Med. 2018 Apr 2;215(4):1187–203. doi: 10.1084/jem.20172246 (PMC5881471; doi:10.1084/jem.20172246)
Supplement: Supplemental Materials (PDF) [file JEM_20172246_sm.pdf]

Supplemental material

Teitz et al., https://doi.org/10.1084/jem.20172246

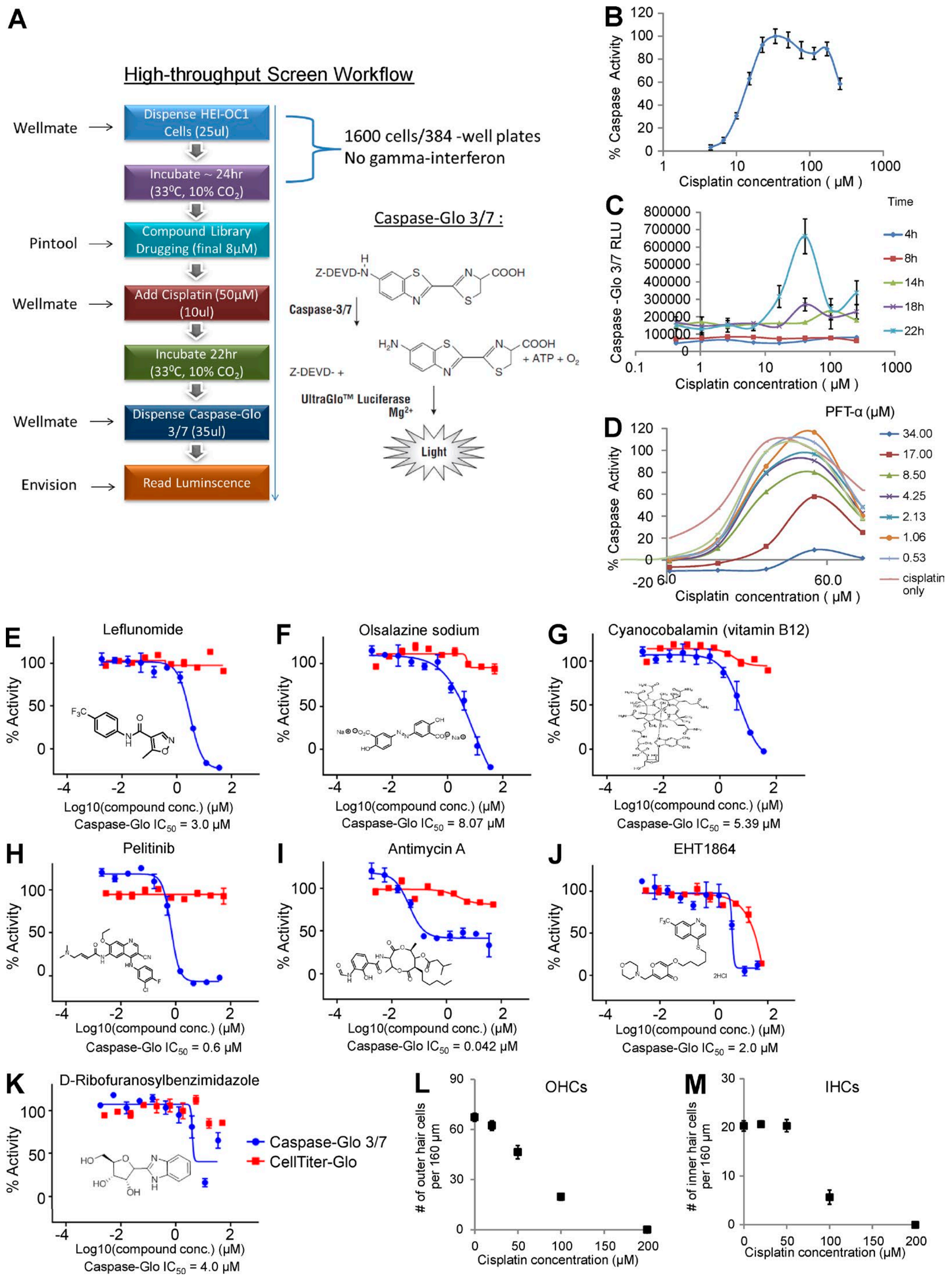

Figure S1. **Outline of the HTS of bioactive compounds, the dose–responses of the top seven hits in the screen in addition to the three CDK2 inhibitors, and cisplatin dose–response curves in the mouse cochlear explants. (A)** HTS workflow. The screen was performed with the help of a robot in 384-well plates after bench optimization. During the screen, HEI-OC1 cells were grown in medium devoid of γ IFN to slow their growth and mimic as closely as possible the conditions of postmitotic nondividing cochlear cells. Caspase-3/7 activity was measured with the Caspase-Glo 3/7 reagent using a substrate that emits luminescence when caspase-3/7 is active. **(B)** Titration of cisplatin concentration (22-h treatment) for use in the screen. We measured caspase-3/7 activity in the screen optimization steps by using increasing cisplatin concentrations. We chose 50 µM cisplatin as the optimum concentration, as it was the lowest concentration to give consistently high caspase-3/7 activity. **(C)** Time course of caspase-3/7 activity in the assay. Caspase-3/7 activity was measured at different time points after the administration of increasing concentrations of cisplatin and was found to peak at 22 h with a cisplatin concentration of 50 µM. Thus, this was chosen as the optimum time point for measuring caspase-3/7 activity in the assay. RLU, relative luminescence units. **(D)** Dose response of the reference compound control in the screen pifithrin-α (PFT-α). This compound was included in each assay plate. The IC_{50} of pifithrin-α in the screen was 17 µM. **(E–K)** Caspase-3/7 activity cisplatin dose responses (blue) and CellTiter-Glo viability dose responses (red) for the top seven hits in the screen (the dose responses of the other three top hits, the CDK2 inhibitors, are shown in Fig. 1). The compounds were tested in triplicate at concentrations ranging from 40 nM to 10 µM, and the cisplatin concentration was 50 µM. The chemical structure of each compound is illustrated. **(L and M)** Cisplatin dose response in P3 or P4 organs of Corti in explant culture (129Sv/C57BL/6 mice). The numbers of OHCs (L) and IHCs (M) per 160 µm are shown. Data are presented as means ± SEM.

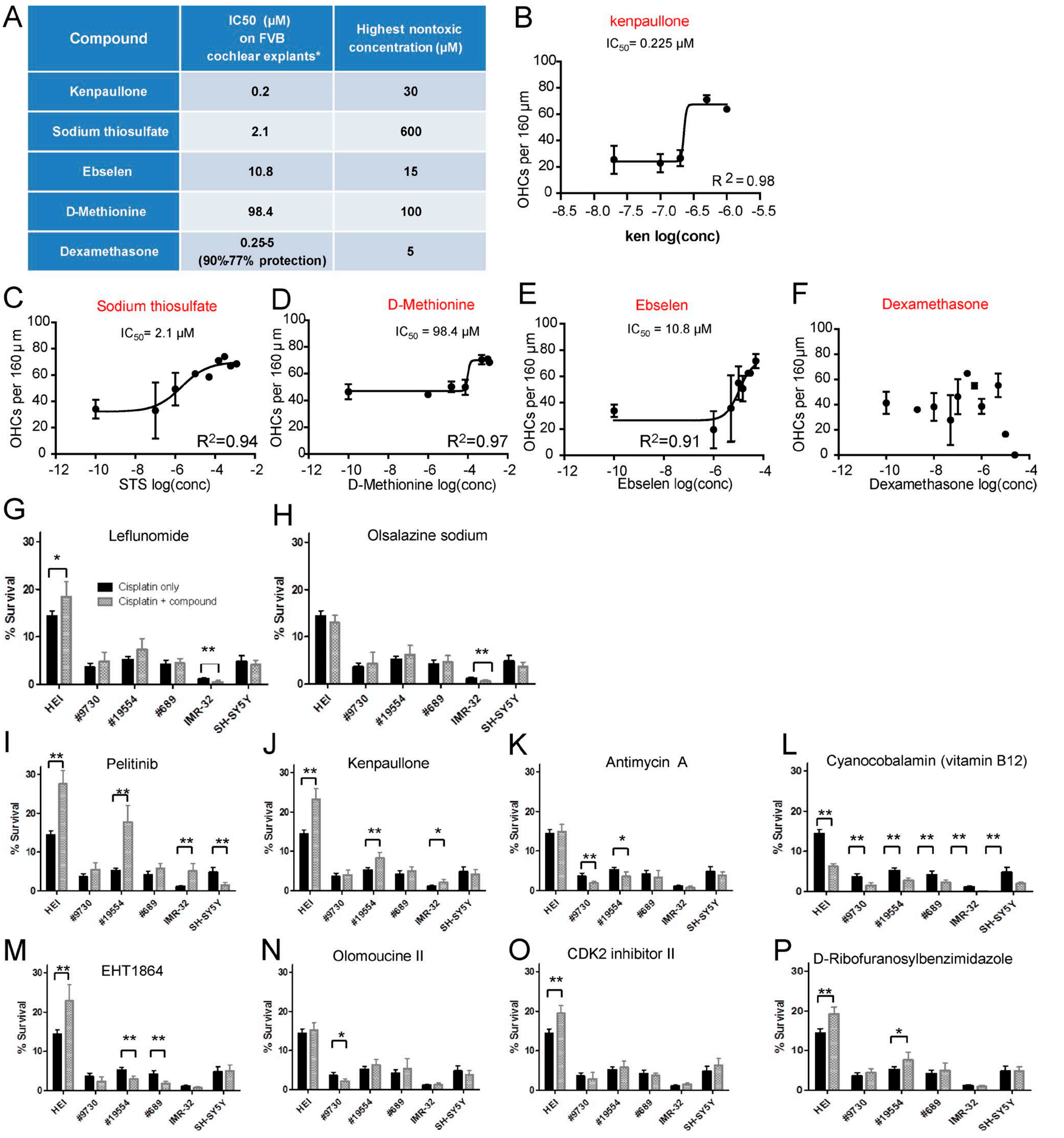

Figure S2. **Comparing kenpaullone with four known benchmark protective compounds in cochlear explants and testing the potential interference of the top 10 hits with cisplatin's killing ability in five tumor lines (MB and NB). (A)** Table comparing the IC_{50} values and highest nontoxic doses of kenpaullone and four other benchmark compounds in FVB cochlear explants. **(B–F)** Dose responses of each tested compound summarized in A. Curves were fitted using Prism, and IC_{50} and R^2 values are shown. Note that dexamethasone showed 77–90% protection at 0.25–5 µM, which cannot be fitted with the Hill curve (F). Mean ± SEM of OHC numbers per 160 µm of middle turn cochleae was shown for each dose. ken, kenpaullone. **(G–P)** Testing the ability of the top 10 compounds to interfere with the cisplatin killing of MB and NB tumors in vitro. The top 10 hits in the screen were tested in a viability assay (CellTiter-Glo; mean ± SEM) after the cells were preincubated for 1 h with the compound at a concentration of three times the EC_{50} for caspase-3/7 in HEI-OC1 cells, followed by treatment for 48 h with or without cisplatin (23 µM) and the test compound (at a concentration of three times the EC_{50}). A cisplatin concentration of 23 µM was chosen for the assay as it is the $EC_{90 \pm 10}$ for all six cell lines, i.e., the HEI-OC1 cells and the five tumor cell lines. The three mouse MB neurosphere cell lines have been described by Morfouace et al. (2014). The two human NB cell lines were IMR-32, which has *N-MYC* amplification, and SH-SY5Y, which does not have *N-MYC* amplification (Barbieri et al., 2006; Altun et al., 2010). *, $P < 0.05$ and **, $P < 0.01$ using Student's *t* test. Kenpaullone (J) significantly protected the inner ear cell line HEI-OC1 but interfered with cisplatin-induced cell death in two of the five tumor cell lines tested and may thus be more suitable for local delivery to the ear. The compound CDK2 inhibitor II (O) significantly protected the inner ear cell line HEI-OC1 and did not interfere with cisplatin-induced cell death in any of the five tumor cell lines, making it more suitable for systemic delivery.

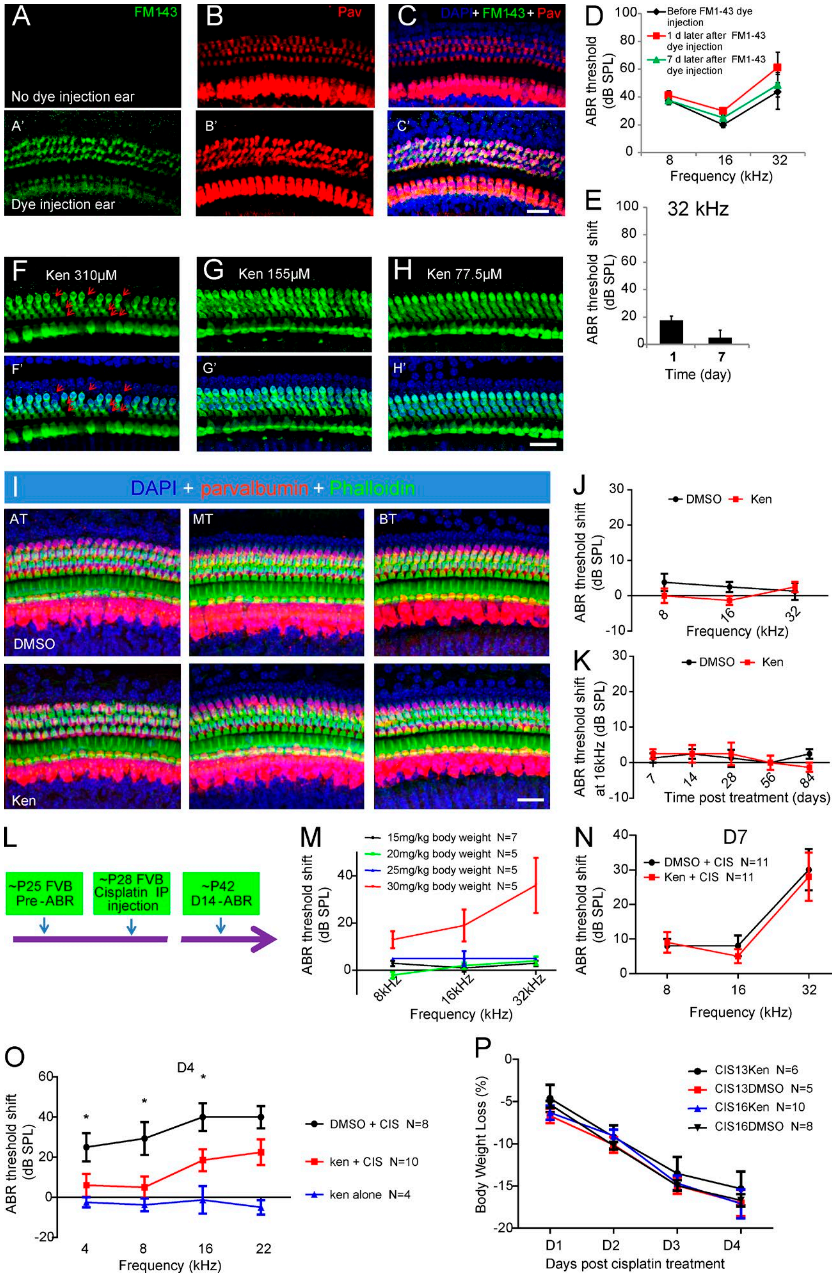

Figure S3. **Local delivery of kenpaullone (ken) in cisplatin (CIS)-treated mice and rats. (A–E)** In vivo tests of trans-tympanic injection of FM 1-43 in adult (P28–P30) FVB mice. (A–C′) Confocal images of apical turns of organs of Corti without (A–C) and with (A′–C′) FM 1-43 dye injection. HCs were labeled after taking up FM 1-43 and costained with the HC marker parvalbumin (Pav) and the nuclear stain DAPI. (D) ABR thresholds before, 1 d after, and 7 d after trans-tympanic membrane injection of FM 1-43. (E) ABR threshold shifts at 32 kHz at 1 d and 7 d after FM 1-43 injection. **(F–H′)** Testing of ken toxicity in vivo in adult FVB mice. Ken at various concentrations (310 µM, 155 µM, and 77.5 µM in 0.5% DMSO) was administered to FVB mice at P28 by trans-tympanic injection. Basal turns of organs of Corti 24 h after ken treatment, as visualized by DAPI (blue) and myosin 7a (green) staining, show that ken is toxic at 310 µM but not at lower concentrations. The arrows indicate lost OHCs. Two mice were tested independently for each dose. **(I–K)** Testing of ken (250 µM) and DMSO (0.5%) long-term toxicity (3 mo after treatment) in vivo in adult FVB mice. Representative images of the organ of Corti are shown in I. The organ of Corti is labeled with phalloidin, parvalbumin, and DAPI. AT, apical turn; BT, basal turn; MT, middle turn. (J) The ABR threshold shifts 3 mo after treatment with DMSO or ken. (K) The time course for the 16-kHz ABR threshold shifts. **(L–N)** In vivo dose responses of CIS in adult FVB mice in three separate cohorts. (L) The experimental design for developing CIS-induced hearing loss in FVB mice. (M) The CIS dose response in the FVB mice at around P28. ABR threshold shifts are shown at D14 after i.p. injection of CIS. (N) Comparison of ABR threshold shifts after D7 for DMSO and ken treatment with CIS damage (30 mg/kg body weight). **(O)** ABR threshold shifts in rats at D4 after treatment with 16 mg/kg CIS. Rats treated with DMSO by trans-tympanic injection and 16 mg/kg CIS i.p. were plotted in black; rats treated with 310 µM ken by trans-tympanic injection and 16 mg/kg CIS i.p. were plotted in red; 310 µM ken alone–treated rats by trans-tympanic injection without CIS treatment were plotted in blue. *, $P < 0.05$ by Student's unpaired *t* test showing significant differences between DMSO+CIS and ken+CIS groups (19.0, 24.4, and 21.6 dB at 4, 8, and 16 kHz, respectively). There were no significant differences between ken+CIS and ken alone groups at these three frequencies. There were no significant differences in threshold shifts between 16 and 13 mg/kg CIS alone–treated rats (Fig. 3 F). **(P)** Accumulative body weight loss (percentage) over 4 d after cisplatin injection in rat groups treated with 13 or 16 mg/kg cisplatin and ken or DMSO. N, number of rats each rat group. Data are presented as the mean ± SEM. Bars, 20 µm.

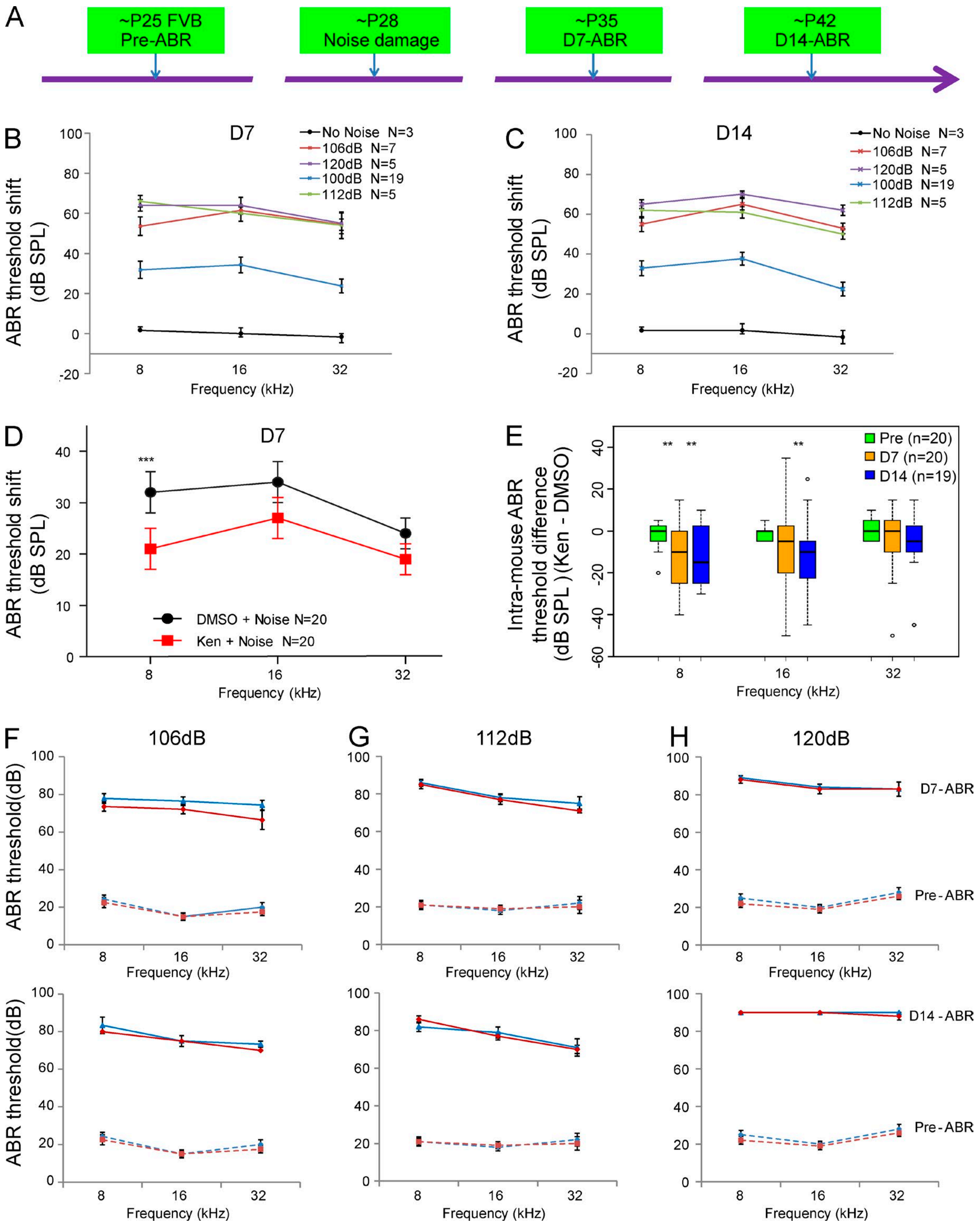

Figure S4. **Local delivery of kenpaullone in noise-exposed mice. (A)** Experimental design in which adult FVB mice at P28 were exposed to noise (8–16 kHz at various noise levels for 2 h). ABR thresholds were recorded before, 7 d after, and 14 d after noise exposure. Cochlear histology was examined at 14 d after exposure. **(B and C)** ABR threshold shifts at D7 and D14 after exposure to noise at 106, 112, or 120 dB, along with measurements for no-noise controls. **(D)** Comparison of ABR threshold shifts 7 d after 100-dB noise damage (D7) with DMSO and kenpaullone (Ken) treatment. ***, P < 0.001 by paired Student's *t* test. **(E)** Box plots of the intramouse ABR threshold differences at 8, 16, and 32 kHz between the two treated ears ($ABR_{ken} - ABR_{DMSO}$) of each mouse before exposure and after exposure at D7 and D14 in 20, 20, and 19 mice, respectively. Significant differences were observed at 8 kHz in D7 and D14 mice and at 16 kHz in D14 mice. **, P < 0.01 by a paired Student's *t* test. Additionally, significant differences were detected by multiple regression longitudinal modeling with time points (before exposure as well as D7 and D14 after exposure) and frequencies (8, 16, and 32 kHz) as explanatory factors (P < 0.01 by a Student's *t* test of regression coefficients). **(F–H′)** Kenpaullone-treated mice showed no protection against hearing loss induced by noise at 106 (*n* = 7), 112 (*n* = 5), or 120 dB (*n* = 5) at 7 d (D7, F–H) or 14 d (D14, F′–H′) after noise damage. Noise damage with DMSO treatment is plotted in blue, that with kenpaullone treatment in red. Data in B–D and F–H are presented as the mean ± SEM.

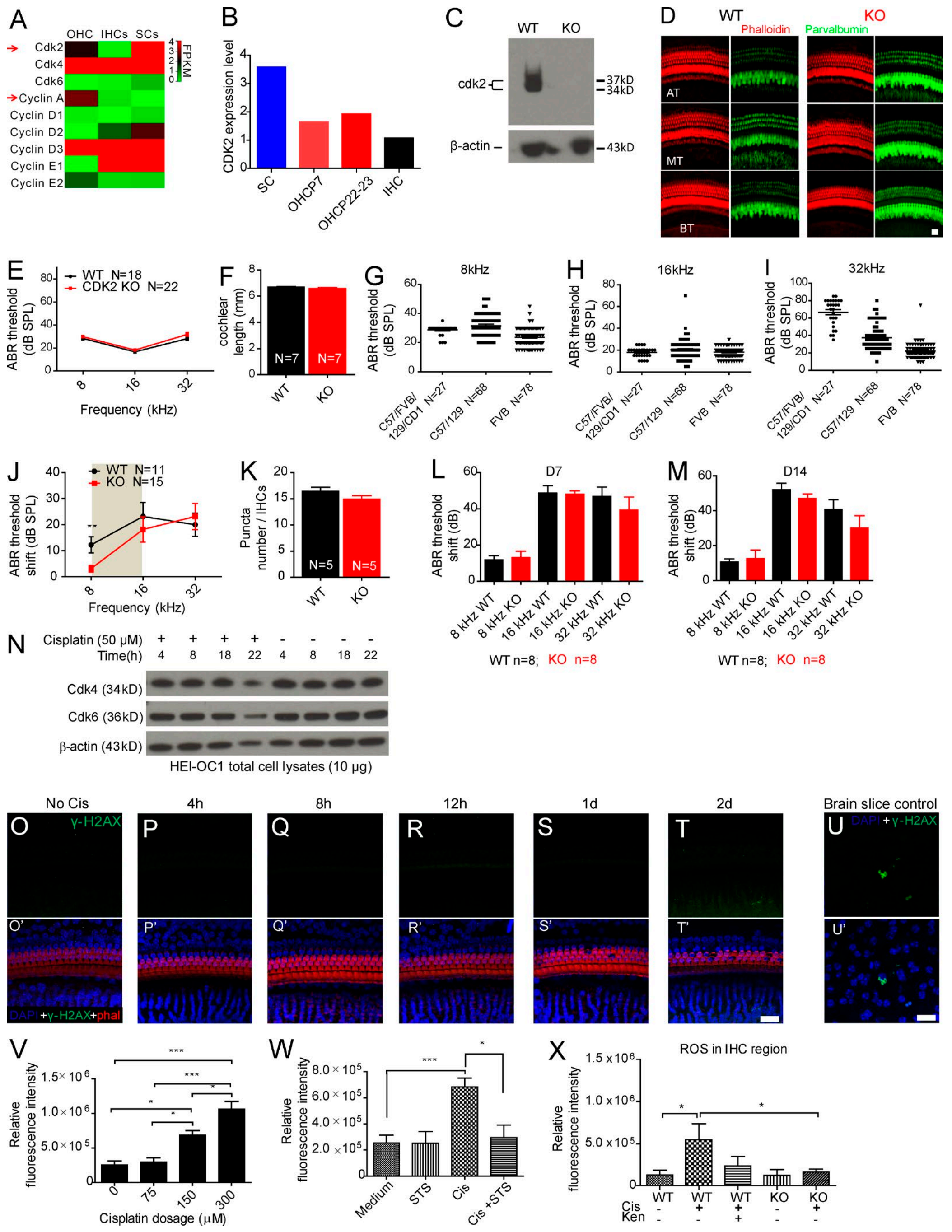

Figure S5. **CDK2 expression in cochleae and CDK2 KO mice in cisplatin- and noise-induced hearing loss; no expression changes were detected in Cdk4, Cdk6 protein levels, or in γ H2AX protein staining after cisplatin treatment. (A)** Heat map analysis of CDK and cyclin gene expression in adult OHCs (P22–P23), IHCs (P74–P75), and SCs (P26–P27). These expression levels are from bulk RNA sequencing analysis of pooled single cells. **(B)** CDK2 expression in the P26–P27 SCs, P7 OHCs, P22–P23 OHCs, and P74–P75 IHCs. **(C)** Immunoblot analysis of total organ of Corti lysates, showing expression of CDK2 protein in WT and CDK2 KO mice. **(D–F)** CDK2 KO mice have normal hearing (E), organ of Corti length (F), and morphology, as determined by phalloidin staining and immunostaining with an antiparvalbumin antibody. **(G–I)** ABR thresholds of adult WT mice on various strain backgrounds before cisplatin treatment, kenpaullone treatment, and noise damage, respectively. **(J)** CDK2 KO protects against hearing loss induced by 100-dB noise damage at D7 after exposure. **(K)** IHC presynaptic puncta counts in the 8-kHz region showed no difference between WT and CDK2 KO mice at 14 d after exposure. Five mice of each genotype were randomly chosen for analysis. **(L and M)** CDK2 KO did not protect against hearing loss induced by 106-dB noise damage at D7 or D14 after exposure. **(N)** CDK4 and CDK6 protein levels did not change during cisplatin treatment (50 µM) in the HEI-OC1 cells. Immunoblots of 10 µg total protein lysates of HEI-OC1 cells with or without 50 µM cisplatin treatment revealed no change in the expression of these proteins during the 22-h time course. β-Actin protein served as the loading control. **(O–T′)** Confocal images of basal turns of organs of Corti after cisplatin treatment (30 mg/kg body weight, for 4 h to 2 d) stained with γ H2AX and DAPI. **(U and U′)** Brain slices were stained with γ H2AX and DAPI as a positive control. **(V)** Cisplatin dose responses of MitoSOX fluorescent intensity in explants. **(W)** Sodium thiosulfate (STS) at 600 µM reduced MitoSOX fluorescent intensity in cisplatin (Cis)-treated explants. Cisplatin concentration used was 150 µM. **(X)** Quantification of ROS signals in IHCs after cisplatin treatment (150 µM) in WT and CDK2 KO mice, as presented in Fig. 6. Bars, 20 µm. Data are presented as the mean ± SEM. *, $P < 0.05$ and ***, $P < 0.001$ by unpaired Student's *t* test.

Table S1. **Top hits for ototoxicity protection**

Compound	Status	IC_{50} (μM) in HEI cells	LD_{50} (μM) in HEI cells	Doses (μM) tested on cochlear explants[a]	Protection (mean % survived OHCs) against cisplatin in cochlear explants (3–5 explants each dose)	Tested doses (μM) and protection (mean % survived neuromast HCs) against cisplatin in zebrafish (5–10 fish each dose)[b]
Antimycin A	In vivo, rodent	0.04	>10	1.8, 0.6, 4 .0	31%, 0%, 0%	Toxic to fish at 0.6 and 1.8
CDK2 inhibitor II	In vitro	0.14	>40	12.0, 1.0, 3.0, 6.0, 12.0	0%, 54%, 100%, 100%, <0%	No protection at 1, 5, 10, 25, 50
Pelitinib	IND phase	0.6	>40	13.0, 6.0, 13.0, 26.0	55%, 49%, 0%, 0%	No protection at 27
Olomoucine II	In vitro	0.8	>40	33, 0.7, 2.0, 6.0, 12.0	0%, 0%, 91%, 73%	No protection at 1, 5, 10, 25, 50
EHT1864	In vivo, rodent	2.0	>20	10.0, 5.0, 10.0	58%, 0%, 55%	No protection at 1
Kenpaullone	In vivo, rodent	2.0	>40	15.0, 0.1, 0.2, 1.0, 5.0, 15.0, 30.0	60%, 0%, 100%, 100%, 100%	30 (30% protection), 50 (50% protection); no protection at 1, 5, 10, 20, 100
Leflunomide	FDA approved	3.0	>40	50.0, 2.0, 10.0, 25.0, 50.0	68%, 0%, 63%, 77%, 100%	No protection at 1, 5
D-Ribofuranosylbenzimidazole	In vitro	4.0	>40	26.0, 5.0 ,10.0, 15.0	0%, <0%, 76%, 0%	No protection at 5, 10, 20, 75
Cyanocobalamin (vitamin B12)	FDA approved	4.2	>10	21.0	53%	No protection at 2.5, 5
Olsalazine sodium	FDA approved	6.2	>40	30.0, 10.0, 59.0	47%, 28%, 0%	No protection at 3, 10, 30

[a]Underlined doses are without preincubation; doses not underlined are with 1-h preincubation with compound alone.
[b]1.5-h preincubation with compound alone.

References

Altun, Z.S., D. Güneş, S. Aktaş, Z. Erbayraktar, and N. Olgun. 2010. Protective effects of acetyl-L-carnitine on cisplatin cytotoxicity and oxidative stress in neuroblastoma. *Neurochem. Res.* 35:437–443. https://doi.org/10.1007/s11064-009-0076-8

Barbieri, E., P. Mehta, Z. Chen, L. Zhang, A. Slack, S. Berg, and J.M. Shohet. 2006. MDM2 inhibition sensitizes neuroblastoma to chemotherapy-induced apoptotic cell death. *Mol. Cancer Ther.* 5:2358–2365. https://doi.org/10.1158/1535-7163.MCT-06-0305

Morfouace, M., A. Shelat, M. Jacus, B.B. Freeman III, D. Turner, S. Robinson, F. Zindy, Y.D. Wang, D. Finkelstein, O. Ayrault, et al. 2014. Pemetrexed and gemcitabine as combination therapy for the treatment of Group3 medulloblastoma. *Cancer Cell.* 25:516–529. https://doi.org/10.1016/j.ccr.2014.02.009
